# Combining Feature-Based Molecular Networking and Contextual Mass Spectral Libraries to Decipher Nutrimetabolomics Profiles

**DOI:** 10.3390/metabo12101005

**Published:** 2022-10-21

**Authors:** Lapo Renai, Marynka Ulaszewska, Fulvio Mattivi, Riccardo Bartoletti, Massimo Del Bubba, Justin J. J. van der Hooft

**Affiliations:** 1 Department of Chemistry, University of Florence, Via della Lastruccia 3, Sesto Fiorentino, 50019 Florence, Italy; 2 Bioinformatics Group, Wageningen University, 6708 PB Wageningen, The Netherlands; 3 Metabolomics Unit, Department of Food Quality and Nutrition, Research and Innovation Centre, Fondazione Edmund Mach (FEM), Via Mach 1, San Michele all’Adige, 38098 Trento, Italy; 4 Department of Cellular, Computational, and Integrative Biology (CIBIO), University of Trento, Via Mach 1, San Michele all’Adige, 38098 Trento, Italy; 5 Department of Translational Research and New Technologies, University of Pisa, Via Risorgimento 36, 56126 Pisa, Italy; 6 Department of Biochemistry, University of Johannesburg, Auckland Park, Johannesburg 2006, South Africa

**Keywords:** human urine, liquid chromatography, untargeted mass spectrometry, computational metabolomics, chemometrics, bioinformatics

## Abstract

Untargeted metabolomics approaches deal with complex data hindering structural information for the comprehensive analysis of unknown metabolite features. We investigated the metabolite discovery capacity and the possible extension of the annotation coverage of the Feature-Based Molecular Networking (FBMN) approach by adding two novel nutritionally-relevant (contextual) mass spectral libraries to the existing public ones, as compared to widely-used open-source annotation protocols. Two contextual mass spectral libraries in positive and negative ionization mode of ~300 reference molecules relevant for plant-based nutrikinetic studies were created and made publicly available through the GNPS platform. The postprandial urinary metabolome analysis within the intervention of *Vaccinium* supplements was selected as a case study. Following the FBMN approach in combination with the added contextual mass spectral libraries, 67 berry-related and human endogenous metabolites were annotated, achieving a structural annotation coverage comparable to or higher than existing non-commercial annotation workflows. To further exploit the quantitative data obtained within the FBMN environment, the postprandial behavior of the annotated metabolites was analyzed with Pearson product-moment correlation. This simple chemometric tool linked several molecular families with phase II and phase I metabolism. The proposed approach is a powerful strategy to employ in longitudinal studies since it reduces the unknown chemical space by boosting the annotation power to characterize biochemically relevant metabolites in human biofluids.

## 1. Introduction

Untargeted tandem mass spectrometry (MS/MS) is one of the most widely used analytical techniques in metabolomics, allowing for the generation of information-rich mass spectral datasets and the identification of metabolic biomarkers in biological complex mixtures [1,2], also thanks to the coupling with separation techniques such as liquid chromatography (LC). Despite the wide application of hyphenated LC-MS/MS platforms, the annotation of biologically relevant metabolites (i.e., biomarkers) is strongly hampered by the complexity of the metabolome and metabolomics data processing and annotation [3]. The annotation process is a pivotal step in untargeted metabolomics that often represents a bottleneck in the process of obtaining biological information and discovering biomarkers. To streamline the metabolite annotation process, metabolomics guidelines have been proposed for the accurate identification and assignment of a metabolite feature [4,5], i.e., through peak picking, mass spectral deconvolution, determination of molecular ions by adduct detection, and fragmentation pattern (MS/MS) analysis [6]. Despite these efforts, the risk of missing relevant information and drawing incorrect conclusions remains relatively high, due to incorrect MS and MS/MS interpretations when matching experimental spectra with available spectral libraries. To aid in structural interpretation, the identification of MS/MS spectral similarities within a given dataset can support the discovery of structurally related metabolites, which plausibly share the same metabolic pathway and/or substructure [7], thus strengthening the biological meaning of the annotations.

In this context, molecular networking (MN) has gained large attention, thanks to the efficient and rapid identification of several molecular families within complex mixtures, providing a visual overview of all the precursor ions, grouped according to their structural relationships, as deduced by their mass fragmentation spectra during an MS/MS experiment [8]. MN uses an unsupervised vector-based computational algorithm to organize molecular ions (i.e., clusters or nodes) into a network of molecular families that share spectral similarities among their MS/MS spectra. At the same time, structural annotation is performed through the Global Natural Products Social Molecular Networking (GNPS) bioinformatics platform [8], which is linked to many mass spectral libraries available as public repository of mass spectra and metadata (i.e., GNPS-MassIVE). Considering the recent growth of public mass spectral libraries, it is expected an increase of the annotation capability (level II or level III) of biologically relevant molecules in comparison with traditional biomarker discovery workflows [9]. MN has been applied in several untargeted LC-MS/MS studies, mainly focusing on phytochemical composition analysis [8], and less frequently on drug metabolism [10], and nutrimetabolomics [11] in human biofluids.

Recently, MN has been extended by its combination with standard feature detection tools into the Feature-Based Molecular Networking (FBMN) workflow that is capable to resolve isomers and incorporate quantitative information (e.g., spectral counts, chromatographic peak areas, etc.), increasing the link between peak picking algorithms and in silico annotation tools [12]. Until now, FBMN has been successfully applied in various fields of metabolomics, allowing level II/level III identification of transformation products of organic micropollutants in water samples [13], native plant constituents [14,15,16], and endogenous urinary metabolites [17]. However, mass spectral library matching is generally performed by the comparison with mass spectral libraries containing MS/MS spectra acquired under a wide range of instrumental conditions (e.g., time-of-flight, orbitrap, hybrid ion traps, etc.) and collision energies used, with different curation protocols providing different mass accuracy levels [13,16], thus suffering from limited reliability of the annotation due to differences in observed mass fragments and their intensity ratios. This issue can be managed by implementing better contextualized libraries containing reference spectra of study-related compounds and acquired under experimental conditions equal to or comparable to the experimental data being analyzed. Finally, FBMN has the hitherto unexploited potential in biomarker research to provide quantitative data of the structurally annotated (and unannotated) features, thus complementing the traditional biomarker discovery procedure with a chemometric protocol that allows establishing their biological significance.

This research investigates the discovery capacity and the extension of the annotation coverage of the FBMN approach, in comparison with a commonly adopted manual annotation of selected significant *m*/*z* features [18]. To this end, the FBMN workflow was applied to deconvoluted and aligned high-resolution LC-MS/MS files of postprandial urine samples from a two-arms intervention study on the intake of *Vaccinium myrtillus* (VM) and *Vaccinium corymbosum* (VC) berry supplements. As far as we are aware, this represents the first nutrimetabolomics application of FBMN to the identification of postprandial endogenous and exogenous metabolites. The MS/MS spectra that were acquired in both negative ionization (NI) and positive ionization (PI) data dependent acquisition modes were compared with the available GNPS libraries. An extensive comparative analysis was done to compare various FBMN parameter settings to arrive at optimal settings for structural annotation purposes in the nutrimetabolomics setting. Furthermore, to support automated nutrimetabolomics annotation workflows, two novel NI and PI contextualized “Nutri-Metabolomics” mass spectral libraries were constructed and made available uniquely on GNPS, each containing MS/MS spectra of about three-hundred food-related human metabolites, acquired under the same mass spectrometric conditions as the study samples. These mass spectral libraries are a fruit of several years of investigations on human responses to dietary interventions at the Edmund Mach Foundation (Italy), and include phase I and phase II human metabolites, as well as food constituents. Special attention was given to microbial metabolites resulting from mixed human and microbiome interaction such as small phenolic acids, phenylacetic acids, phenylpropionic acids, indoles, and carbolines, as well as bile acids. Other classes include sulfate and glucuronides conjugates of common food constituents such as caffeic acid glucuronide, dihydroferulic acid sulfate, isoferulic glucuronide, etc. Several aroma compounds were included to facilitate substructure matching, as those were observed in biological fluids in conjugated form (monoterpenoids, safranal, furfuran, fenchyl alcohol etc.). Finally, the spectral library offers specific advanced glycation end-products including pyrraline, furosine and more.

In the current study, the mass spectral library creation aimed at increasing (i) the accuracy in the annotation thanks to a better match of instrumental metadata such as detector and collision energy, (ii) nutrimetabolomics knowledge on postprandial analysis of biological samples and plant-based food intake. Additionally, the quantitative data within NI and PI FBMN networks were exploited to gain insights into (i) metabolites characterized by different postprandial kinetics and (ii) the relative dietary contribution of VM and VC interventions of the identified metabolites.

## 2. Materials and Methods

### 2.1. Chemicals and Reagents

Full purchase details of solvents and standards used are reported in Section S1 of the Supplementary Materials. The complete list of the reference standard adopted to build the “Nutri-Metabolomics” libraries, in both NI and PI modes, is shown in the List of Reference Standards used to build the libraries.xlsx file in the Supplementary Materials.

### 2.2. Study Design, Sample Extraction, and LC-MS/MS Analysis

The datasets analyzed in this research are part of a more comprehensive clinical intervention trial, based on the hematic and urine biomarker discovery on the intake of VM and VC [18,19]. Urine samples of each volunteer (*n* = 10 for each intervention) were collected at baseline and 30, 60, 120, 240, and 360 min after VM or VC supplement intake. Pooled urine samples were also collected 24 h and 48 h after supplement intake. Details of supplements characterization (Table S1), as well as study design are reported in Section S2 of the Supplementary Materials. Urine samples were extracted and analyzed as reported elsewhere [19]. The entire procedures of extraction and LC-MS/MS analysis of urine samples are reported in Sections S3 and S4 of the Supplementary Materials, respectively. The entire sample set was acquired in full scan mode, collecting high quality data for an appropriate statistical analysis, as well as in data dependent acquisition (DDA) mode, to leverage large quantities of MS/MS data for structural investigation preserving the kinetic heritage of the study design.

### 2.3. Data Pre-Processing

The full scan files were processed and analyzed as previously reported [18]. Additionally, the data-dependent spectra files (including blanks) were converted from the *.raw* to *.mzML* MS convert by ProteoWizard (https://proteowizard.sourceforge.io) (accessed on 15 April 2021). Further data processing was performed with MZmine 2 software [20], separately for NI and PI datasets. Data pre-processing included the following steps: mass detection, chromatogram reconstruction and deconvolution, isotope grouping, alignment and gap filling. Subsequently, the aligned feature lists were exported as MS/MS files (.mgf format) and quantification tables (.csv format of aligned features and related chromatographic peak areas), according to GNPS documentation on FBMN (https://ccms-ucsd.github.io/GNPSDocumentation/) (accessed on 21 April 2021).

### 2.4. Data Availability: MassIVE Repository, Metadata and GNPS Jobs

Data in *.mzML* format are available on-line on GNPS infrastructure (MSV000088336). The metadata describing file/sample properties were entered manually for all samples and organized in two different files according to the acquisition polarity of the uploaded MassIVE datasets, following the GNPS guidelines (https://ccms-ucsd.github.io/GNPSDocumentation/metadata/) (accessed on 21 April 2021). In detail, metadata consisted of three descriptive categories, (i) spectrum file name (the same of acquired raw data), (ii) type of supplement (VM or VC), and (iii) related time point after intake. These elements are required to get a correct grouping within FBMN for quantitative analysis (see the Metadata and Library Information.xlsx file in the Supplementary Materials). For the upload on GNPS, metadata files were converted to *.tsv* files. The FBMN analysis are available at the following links:

- PI: https://gnps.ucsd.edu/ProteoSAFe/status.jsp?task=a981ebd40809453ebe1524ff1fc8e265 (accessed on 27 June 2021).
- NI: https://gnps.ucsd.edu/ProteoSAFe/status.jsp?task=0a239e71bb2045c292c4c96f4501249c (accessed on 27 June 2021).

### 2.5. “Nutri-Metabolomics” Library Building and Implementation

The analytical standards used to build the in-house libraries were acquired in the same MS/MS conditions as study samples (replicated three times), which are reported in Section S4 of the Supplementary Materials. GNPS provides a platform to build MS/MS spectral libraries, requiring good quality MS/MS spectra and annotation spread sheets containing key and machine-readable descriptors such as file name, compound name, SMILES, InChiKey, PubMed. To build the library, only pure analytical standards were used, thus no putative or un-known compounds are present in the files. Two annotation spread sheets were built in NI and PI, containing 319 and 339 injected compounds, respectively (see the Metadata and Library Information.xlsx file in the Supplementary Materials). Analysis of standards included their separation on the chromatographic column; however, a retention time match is not supported in GNPS and therefore this information was used manually when needed. The “Batch Validator Workflow” [21] step was run to evaluate the correct match between spreadsheets (dropped as .csv files), and original spectra. The completed libraries can be found in the public spectral library collection of GNPS named as “Nutri-Metabolomics”.

### 2.6. Molecular Networking Analyses

Molecular networks were obtained following the online workflow on the GNPS web-platform (https://gnps.ucsd.edu/) (accessed on 21 April 2021). FBMN was performed adopting the most suitable basic and advanced networking options, selected through the recommended network qualitative optimization by classical MN (see Section 3.1), for NI and PI dataset exported from MZmine 2 software. The detailed investigation of MN options is reported in Section S5 of the Supplementary Materials. The most appropriate input parameters were set as follows: NI were analyzed using precursor ion mass tolerance (PIMT) and fragment ion mass tolerance (FIMT) equal to 0.1 Da and 0.01 Da, respectively. The other parameters were set as follows: minimum matched fragment ions = 3, networking cosine score > 0.6, library cosine score > 0.5, and minimum library shared peaks = 3. PI dataset was processed adopting PIMT = 0.05, FIMT = 0.05, minimum matched fragment ions = 3, networking cosine score > 0.5, library cosine score > 0.3, and minimum library shared peaks = 3. Network analysis and quantitative results were investigated and exported adopting Cytoscape environment [22]. Moreover, unknown nodes were annotated with putative molecular structures by manual annotation based on: (i) mass difference between identified and unknown node, (ii) precursor ion mass accuracy, and (iii) fragmentation patterns in MS/MS spectra (see Section S6 of Supplementary Materials).

### 2.7. Analysis of Postprandial Kinetics

Reinjection of the entire dataset in DDA fashion enabled the exploitation of postprandial kinetics data. To extract the postprandial information from FBMN, the Pearson product-moment correlation (PPMC) analysis was performed, using the “corrplot: A visualization of a correlation matrix” package implemented in R (https://cran.r-project.org/) (accessed on 28 July 2021), thus estimating the linear correlation between the mean chromatographic peak area of identified nodes and time points. The quantitative FBMN data used for the correlation analysis were extracted from the “node table” of the Cytoscape environment, built using the loaded metadata for both NI and PI datasets. Statistically significant (*p*-value ≤ 0.05) PPMC coefficients (r) were used to discriminate early (1–2 h postprandial) from late (approximately 4 h and more postprandial) occurring postprandial metabolites, which are commonly considered as the result of phase II or phase I metabolism, respectively [23]. Accordingly, positive and negative r-values indicated nodes associated to phase I (late postprandial) and phase II (early postprandial) metabolism, respectively. A limitation of using PPMC within FBMN was the absence of sample normalization as this functionality is currently not available. Findings from this step were compared to those obtained through the PPMC analysis of longitudinal variations of the chromatographic area of aligned features (i.e., outside FBMN), as a control strategy. It should be highlighted that, although the PPMC coefficients can be associated with the metabolism phase, its relation to the specific food intake remains elusive without further biochemical interpretations. Simultaneously, full scan data underwent the conventional data processing, as previously described [17]. Briefly, biomarkers of food intake in postprandial responses were selected by applying selected R packages to full scan data [24], according to the following two-step procedure: (i) verification of increasing trend along time points and (ii) calculation of AUC curves and intra-intervention discrimination. Statistically significant features were annotated manually with use of on-line spectra databases such as mzCloud and HMDB. Details of this procedure are reported in Section S5 of Supplementary Materials.

## 3. Results and Discussion

**Figure 1:**
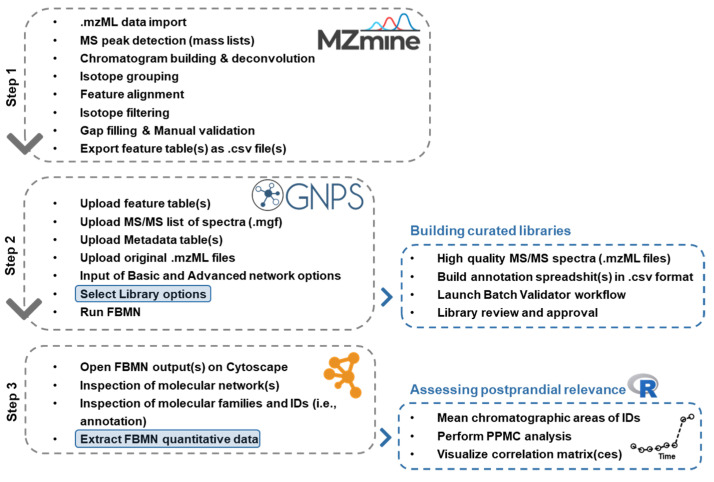
Schematic representation of the available (grey dashed lines) and proposed (blue dashed lines) workflows of data management, applied by the combination of Feature-Based Molecular Networking and the “Nutri-Metabolomics” mass spectral libraries that were manually curated.

The NI and PI datasets were treated following the workflow illustrated in Figure 1, which integrates the PPMC analysis of postprandial kinetics within the FBMN environment. However, since FBMN extracts only the mean values of the chromatographic area as quantitative data for PPMC analysis, thereby losing knowledge of inter-individual variability, the variance of metabolite feature abundance among volunteers was investigated at each time point as a control, before applying the FBMN workflow. Accordingly, the coefficient of variation (CV%) of chromatographic areas of each aligned feature within a same time point was calculated, highlighting a strong variability (CV% approximately in the range of 30–300% and median higher than 100% in most cases). These findings highlighted the importance of evaluating the results of PPMC analysis at the population level.

### 3.1. Optimization of the Input Parameters for Network Analysis

Before running FBMN, various networking basic and advanced options must be investigated to find out the most suitable parameters to perform the MN analysis. To properly evaluate the effect of input parameters, the total number of nodes (precursor ions with identical fragmentation pattern, i.e., consensus spectrum), edges (i.e., node connections related to structural similarities), identified compounds (IDs, i.e., annotated through spectral library matching), and spectral families (i.e., the groups or clusters, also referred as molecular families), were analyzed in both NI and PI datasets and the results are reported in Figure S1 of the Supplementary Materials. In this regard, increasing PIMT value, the number of nodes, edges, and spectral families decreased, whereas the number of IDs showed a predominantly increasing trend, mainly due to the less strict conditions as consensus spectra got merged (i.e., considering different isobaric compounds as one) at increasing PIMT. Hence, to keep a reliable number of nodes and spectral families without significantly affecting the number of IDs, PIMT was set at 0.1 Da and 0.05 Da for NI and PI, respectively. FIMT exerted an effect on the output variables like that of PIMT, except for the total number of edges, which increased by increasing values of FIMT. Due to the loss of accuracy in node networking for high FIMT, values of 0.01 and 0.05 were chosen for NI and PI datasets, respectively. The number of minimum matched fragment ions was set at 3 for both NI and PI for two reasons: (i) Its increase exerts a significant reduction of the number of nodes with an ID and their reliability, (ii) many food-derived metabolites have only a few characteristic mass fragments. Cosine scores for networking and library matching affected mainly the number of spectral families and of IDs, respectively. A good compromise between these two outputs was obtained by setting the networking and library matching cosine score thresholds at 0.6 for NI and 0.5 for PI. Finally, the number of minimum library shared peaks was set at 3, because higher values of this parameter were responsible for a drastic reduction of IDs, similarly to what was observed for the number of matched fragments.

### 3.2. FBMN Annotation of NI and PI Datasets

FBMN workflow applied to NI and PI datasets combined with aligned feature lists and quantitative tables exported from data pre-processing, was able to remove the 57% and 27% of NI and PI redundant IDs (i.e., artefacts like duplicated features) found by classical MN, respectively.

As first result, the effect of including context specific “Nutri-Metabolomics” mass spectral libraries in the annotation workflow was evaluated by applying the FBMN protocol in their presence and absence (i.e., GNPS libraries “only”). Indeed, substantial advantages were observed upon using the dedicated mass spectral libraries, i.e., the increase of (i) 20%, 48%, in the number of IDs (Figure S2A) and (ii) 62.5%, 34%, in the number of IDs with a mass error < 5 ppm (Figure S2B), for NI and PI datasets, respectively. Additionally, the use of the “Nutri-Metabolomics” libraries solved two mis-annotations (i.e., incorrect annotation of nodes) in the NI datasets. These results highlight the importance of applying the FBMN annotation strategy in combination with contextual libraries, i.e., containing true reference standards that are relevant for the application of interest and analyzed under the same instrumental conditions adopted for the analysis of real samples.

The FBMN network of the NI dataset consisted of 545 nodes and 799 edges, with a total number of connected components equal to 307, corresponding to 65 spectral families, whereas molecular networking of the PI dataset resulted in 5079 nodes and 6904 edges, with a total number of connected components equal to 3543 (i.e., 663 spectral families). The ID lists obtained from the library matching in both NI and PI datasets contained 39 and 384 unique annotated compounds, respectively, which were checked for mass accuracy to be around or lower than 5 ppm. Table S2 (see Section S5 of the Supplementary Materials) reports the metabolites identified by library matching (based on cosine score similarity) of nodes within and outside molecular families (the latter are typically called singletons) from both NI (24 IDs) and PI (43 IDs) datasets, characterized by the lowest mass error (∆ ppm).

Even though the FBMN approach has specific methodological inputs and results that differentiate it from commonly used workflows in untargeted nutrimetabolomics, it is interesting to compare the discovery capacity and annotation coverage obtained with other approaches. For this purpose, the FBMN was compared against two widely used annotation protocols: (i) MZmine Library Search and (ii) statistical-based feature selection followed by manual annotation (see Section S5 of the Supplementary Materials) [18]. It should be emphasized that the compared workflows differ substantially as per their rationale. MZmine Library Search workflow matches each row of the NI and PI feature lists (used also for FBMN) against the imported spectral library. To make a consistent comparison with the annotation performed with FBMN, the “ALL-GNPS” library was used. The conventional protocol aims at selecting only statistically significant *m*/*z* features from full scan data, followed by manual annotation using the MS/MS spectra often obtained in targeted mode. In contrast with the presented approaches, FBMN explores all available MS/MS data from the DDA metabolomics profiles (taking advantage of all structural annotations that can be made), annotating them against mass spectral libraries. Only then, further statistical analysis is performed to discover their potential postprandial relevance. Thus, the direct comparison of these annotation and prioritization workflows is not and will never be straightforward; yet, here we highlight some relevant aspects.

Table 1 shows the final number of IDs found adopting the three approaches. MZmine Library Search workflow provided the metabolite annotation with 26 and 49 unique IDs in NI and PI datasets, respectively, with cosine similarity scores (isotopic pattern at full scan level) higher than 0.7. The number of IDs identified by this approach was comparable with the results of the applied FBMN workflow, and several metabolite categories were commonly annotated by the two procedures (data not shown), such as hippuric acids, catechols, and derivatives of phenylacetic acid, coumaric acid, indoles, and hydroxybenzoic acid. However, due to the format of our data unsuitable for MS/MS-based mass spectral matching within MzMine (i.e., incomplete mass lists for the MS/MS scans), the MZmine-based approach relied on precursor *m*/*z* and isotope pattern matching, thus possibly resulting in a less reliable annotations due to the limited structural information. The statistical-based/manual annotation method resulted in 50 and 106 statistically significant *m*/*z* features in PI and NI datasets, respectively, corresponding to 24 metabolite features after manual checking. Manual structure elucidation putatively identified 18 metabolites (12 in NI and 6 in PI datasets), while 6 metabolites remained unknown (see Table S3). Using FBMN, a higher number of metabolites was putatively annotated, i.e., 24 IDs in NI, and 43 IDs in PI, when compared to the statistical-based/manual annotation approach. These differences were due to both (i) the automatic query (intrinsic of FBMN) of all publicly available mass spectral libraries, including “Nutri-Metabolomics” ones, and (ii) the different strategies to select the metabolite features to be annotated. In fact, the conventional approach processes the NI and PI datasets to highlight physiologically relevant features, before their annotation is performed by unqueried matching with analytical standards available in on-line spectral libraries. On the contrary, FBMN automatically generates a list of IDs, which is then refined by applying, for example, a mass accuracy threshold, in combination with the use of mass spectral similarity scoring (i.e., modified cosine score), as presented in this study. Despite these methodological differences, hydroxyhippuric acid and dihydrocaffeic acid glucuronide were identified with both approaches. Moreover, the conventional postprandial analysis confirmed the FBMN identification of structurally-related metabolites significantly altered upon berry intake, belonging to furoic and abscisic acid derivatives, hydroxy and/or methoxy benzoic acids. By contrast with FBMN, the conventional protocol for postprandial analysis identified the metabolite categories of valerolactone and valeric acid derivatives (see Section S5 and Figure S3 of the Supplementary Materials), which are well-known colon-derived catabolites of flavanols [25]. These metabolite features were found also inside the FBMN molecular networks; however, they were not structurally characterized as such, due to their absence in the “Nutri-Metabolomics” and other mass spectral libraries. These findings highlighted the importance of expanding the coverage of online spectral repositories to boost metabolite annotations.

### 3.3. VM and VC Relative Contributions to the Postprandial Metabolome

Categorization of NI and PI metadata based on VM and VC interventions (see Section 2.4 for details) allowed for the separate storage of spectral counts (i.e., the number of mass spectra recorded for a node) of each ID precursor ion. This information was used here for assessing the VM and VC relative contributions of each ID to the postprandial metabolome, by the representation of a pie chart (see Figure 3 and Figure 4 in Section 3.5). Moreover, a preliminary and descriptive contribution to the annotated urinary metabolome of VM and VC interventions can be estimated. Interestingly, VM and VC interventions exhibited an opposite feature occurrence in the two ionization datasets, highlighting the importance of investigating both polarity modes. In detail, NI IDs resulted in a higher postprandial occurrence after the intake of VC supplement (62 ± 6% vs. 38 ± 4% for VC and VM), whereas for PI dataset, a slight predominance was found for VM (54 ± 2% vs. 46 ± 2% for VM and VC).

### 3.4. PPMC Analysis of Postprandial Kinetics

**Figure 2:**
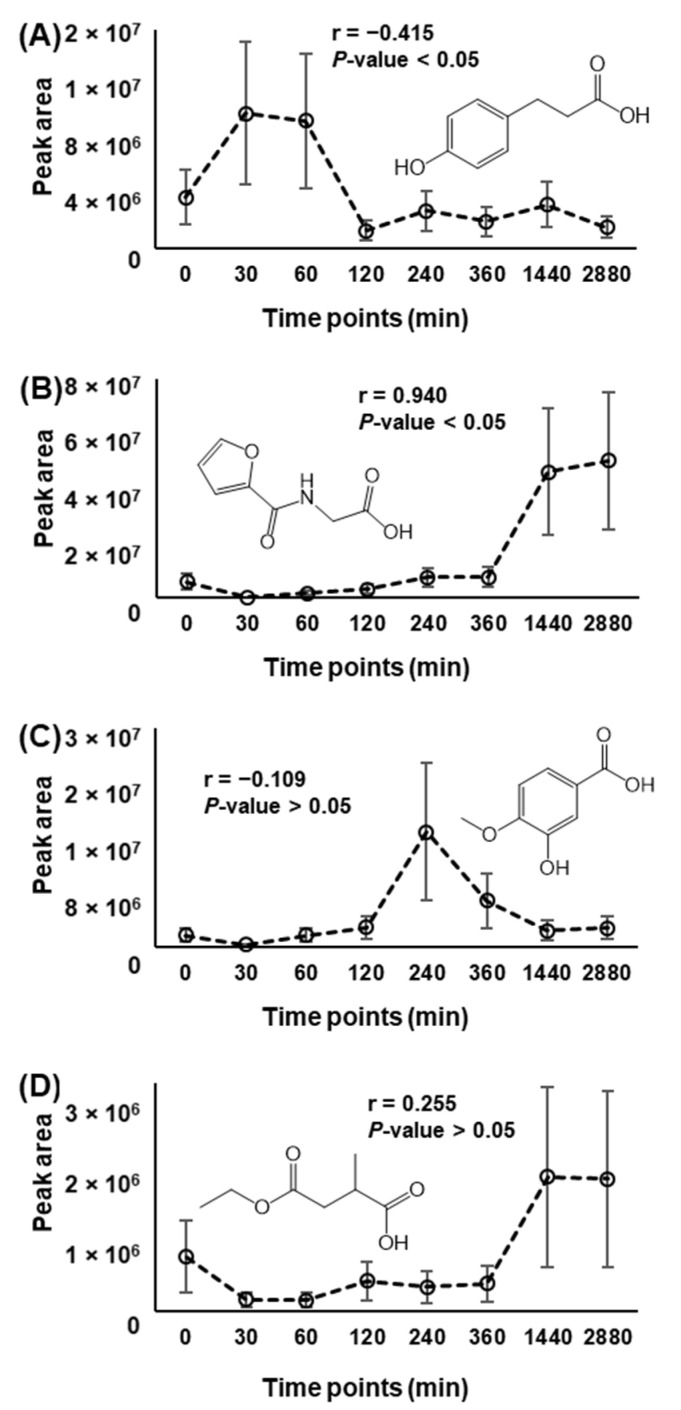
Representative examples of the use of PPMC coefficients (r) to evaluate phase I and II metabolisms of annotated metabolites (Table S2), as described in Section 2.7 of the main text. (**A**) Significant and negative r-value indicating a phase II metabolism. (**B**) Significant and positive r-value indicating a phase I metabolism. (**C**) Non-significant and negative r-value. (**D**) Non-significant and positive r-value.

Longitudinal data analyzed by FBMN approach allows for additional data exploration to highlight the specificity of food intake as well as the “background” metabolism, since no feature selection is performed. Accordingly, the PPMC analysis was performed on the mean values of chromatographic area of each ID as a function of time. Following this analysis, 65.7% of the annotated metabolites (i.e., 44 IDs on a total of 67) showed a statistically significant trend approximating an increasing or decreasing postprandial response, thus highlighting the reliability of this approach. Among the significant correlated metabolites, 35 IDs showed a positive coefficient (r-values) and were therefore associated to phase I metabolism, whilst 9 IDs were characterized by negative r-values, suggesting a phase II metabolism. Figure 2A,B illustrates two representative postprandial trends of IDs corresponding to significant negative and positive r-values, respectively.

The remaining 23 metabolites exhibited a non-linear and not significant trend, as shown in the two representative examples of Figure 2C,D. The postprandial behavior of these IDs cannot therefore be assigned through this approach and requires a qualitative investigation (plots of chromatographic areas vs timepoints) and/or a dedicated treatment outside the FBMN environment.

As stated above, these results were based on the correlation analysis of mean values of chromatographic areas, i.e., without considering the dispersion of individual data around the mean. To evaluate the impact of the extent of this dispersion on the statistical significance of linear correlations, the data obtained for each volunteer and for each annotated feature were submitted to PPMC analysis outside the FBMN environment (i.e., using the data from MZmine feature lists). For 25 IDs out of the 44 IDs found to be significant based on mean chromatographic areas, the statistical significance of the r-values was confirmed, notwithstanding the high variability observed in the peak area datasets. These results encourage the applicability of the postprandial analysis proposed here at least as a first immediate screening of the postprandial behaviour of annotated metabolites, capturing their metabolic trends over time. It is of note that this approach would produce more accurate results when a lower dispersion of individual data around the average value is observed; and to achieve this, increasing the sample size may be of help.

### 3.5. Nutrimetabolomics Outcomes from FBMN Molecular Networks

Figure S4 of the Supplementary Materials shows representative examples of the structural modification involved in phase I and II metabolism of well-known VM and VC native constituents [26], in association with the annotated metabolites and their significant PPMC r-values.

Accordingly, potential metabolic modifications such as conjugations (e.g., glucuronidation) and additions (e.g., methylation) should be expected to undergo in-source hydrolysis and dissociation, leading to accurate annotations, but losing a relevant structural information. To limit these drawbacks, a robust network inspection was performed to ensure a reliable annotation.

**Figure 3:**
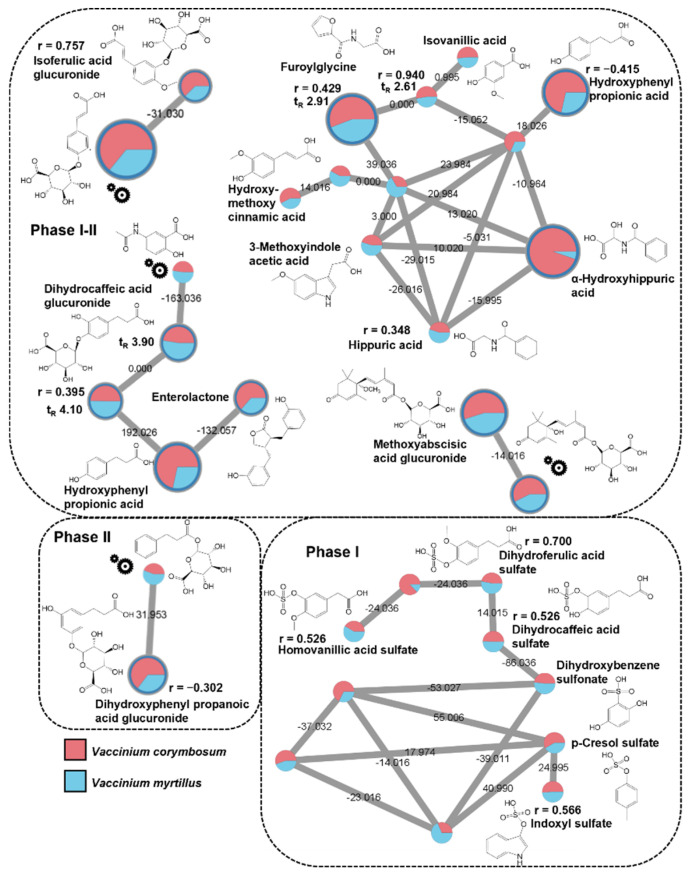
Extracted molecular families of identified metabolites in negative ionization mode listed in Table S2, belonging to the category of (poly)phenolic compounds, abscisic acid, and their glucuronide or sulfate derivatives. Dashed boxes group the identified metabolites according to phase I and II metabolism following PPMC analysis. The “gear” symbols refer to the putative structure identified by manual investigation as reported in Section 2.6 of the main text. Statistically significant Pearson correlation coefficients (r) are reported. Edge labels refer to the mass difference between two nodes.

Within NI dataset (24 IDs, see Table S2), four singletons were identified through spectral matching with a good mass accuracy: azelaic acid, galacturonic acid, glutamine, and ethoxy-oxobutenoic acid. The occurrence of galacturonic acid and glutamine can be addressed to in-source dissociation of glycosidic and peptidic bonds of metabolite conjugations. Figure 3 illustrates the molecular families in which at least one of the remaining 20 metabolites was annotated. These metabolites were grouped according to their postprandial kinetics, as assessed by statistically significant r-values. In Figure 3, the structure of unknown nodes labelled with a gear was proposed as level III identification by the analysis of their MS/MS spectra the hypothesized scheme of fragmentation (Figure S5 of the Supplementary Materials).

About the 50% of the identified structures was characterized by molecular scaffolds related to cinnamic and dihydrocinnamic acids. Interestingly, among unknown nodes, a relevant number of putative glucuronide derivatives was easily recognized by the occurrence in the MS/MS spectra of peaks at *m*/*z* 175.02 and 113.02, typical of glucuronic acid (Figure S5).

Two nodes highlighted in one box in Figure 3 were recognized as a molecular family related to abscisic acid glucuronide derivatives. The ID occurring in this family, was at first addressed as dihydroxy-diphenylphenoxy-trihydroxyoxane-carboxylic acid, with a mass error of about 128 ppm. However, the inspection of its MS/MS spectra (see Figure S6 of the Supplementary Materials) led to a more accurate putative annotation of this node as methoxyabscisic acid glucuronide (Δ = 2.6 ppm). In addition, the hypothesized structure of the linked node was consistent with abscisic acid glucuronide (Figure S5), which was already putatively identified in a previous study by a conventional annotation workflow [18].

Among the identified molecular families in the NI dataset, some of them exhibited a mixed metabolic contribution (i.e., phase I–II). In detail, isoferulic acid glucuronide showed a positive and significant PPMC correlation (r = 0.757), but was linked with a node exhibiting an opposite postprandial behavior (peak area vs. time points, data not shown), thus suggesting a phase I-II mixed contribution.

An analogous mixed metabolic contribution can be also proposed for the abscisic acid spectral family since the methoxyabscisic acid glucuronide is most likely associated to phase I due to the methylation of the hydroxyl group, whereas the node putatively associated to the glucuronide derivative of abscisic acid, is related to phase II.

The molecular family containing hydroxyphenyl propionic and hydroxy-methoxy cinnamic acids was characterized by peculiar structural relationships and depicted a heterogeneous metabolic contribution. In fact, the postprandial analysis of the identified nodes evidenced a phase I expression (0.348 < r < 0.940) for most metabolites [19,27], with the only exception of hydroxyphenyl propionic acid, for which a phase II metabolism can be suggested, based on its r-value (−0.415). Even though this metabolic association to phase II metabolism is apparently questionable due the lack of a conjugated group, the analysis of the full scan spectra evidenced an in-source fragmentation of the sulfate derivative of the hydroxyphenyl propionic acid (*m*/*z* = 263.02 Da), thus confirming the phase II metabolic attribution. A detailed analysis of this molecular family evidenced also that the compound annotated as hydroxy-methoxycinnamic acid probably underwent in-source dissociation, since in the same t_R_ range, an ion at *m*/*z* 273 fragmented in *m*/*z* 229.02 and *m*/*z* 193.05, corresponding to losses of 44 Da (neutral loss of CO_2_) and 80 Da (loss of SO_3_). These findings suggested that the annotated compound was conjugated with sulfate. The other spectral family associated with the phenylpropionic scaffold also included metabolites associated with both phase I (i.e., enterolactone and hydroxyphenylpropionic acid) and phase II (i.e., dihydrocaffeic acid glucuronide) [28]. A clearer postprandial kinetics was highlighted for the molecular family of dihydroxyphenyl propanoic acid glucuronide, being addressed as phase II (r = −0.302) metabolites [26]. Several sulfate metabolites occurred in the same molecular family, belonging to the categories of dihydrocinnamic and vanillic acids, phenolic derivatives, and indoles. Thanks to the analysis of postprandial profiles and in agreement with literature findings [29,30], the molecular scaffolds of the identified molecules are probably related to the activity of the gut microbiota. The metabolites occurring in this spectral family can be addressed to the phase I metabolism (0.526 < r < 0.700). Ultimately, it should be emphasized that some IDs belonging to the abovementioned molecular families showed structural similarities with previously annotated compounds. For example, compounds 4 and 5 of the NI dataset (Table S2), are characterized by retention time and MS2 fragments like those reported for the related glucuronidated conjugates found in urine by Ancillotti and co-workers [19].

In the PI dataset, twenty-four singletons were identified. In detail, several metabolites, annotated as (poly)phenolics and phenolics derivatives, were linked to phase I metabolism (e.g., dihydroxy-trimethyl-isochromenone, trihydroxybutyrophenone, and dihydroresveratrol) and with mixed contribution of phase I-II (e.g., cinnamic acid and hesperetin) by PPMC analysis. Other plant endogenous compounds, annotated with high accuracy, did not show any significant PPMC. Among them, β-glucopyranosyl-tryptophan and furaneol, as well as abscisic acid and nerol, which are well-known food-intake biomarker [31,32], and plant constituents [33,34], respectively. Some human endogenous compounds were also annotated (i.e., alpha-CEHC, ethylindole carboxylicacid, folinic acid, formylkynurenine, indole acetic acid, sebacic acid, ketodeoxycholic acid, keto-octadecadienoic acid, and hydroxy-methoxybenzophenone), exhibiting different trends against time points (−0.645 < r < 0.958), thus resulting in a complex metabolic output potentially associated with the investigated interventions, or resulting from background diet. Finally, PI mode exhibited three singletons that matched the NI annotations (i.e., azelaic acid, furoylglycine and enterolactone) and postprandial behavior interpretation based on PPMC analysis, being their longitudinal trend characterized by high and positive r-values (0.640 < r < 0.967).

**Figure 4:**
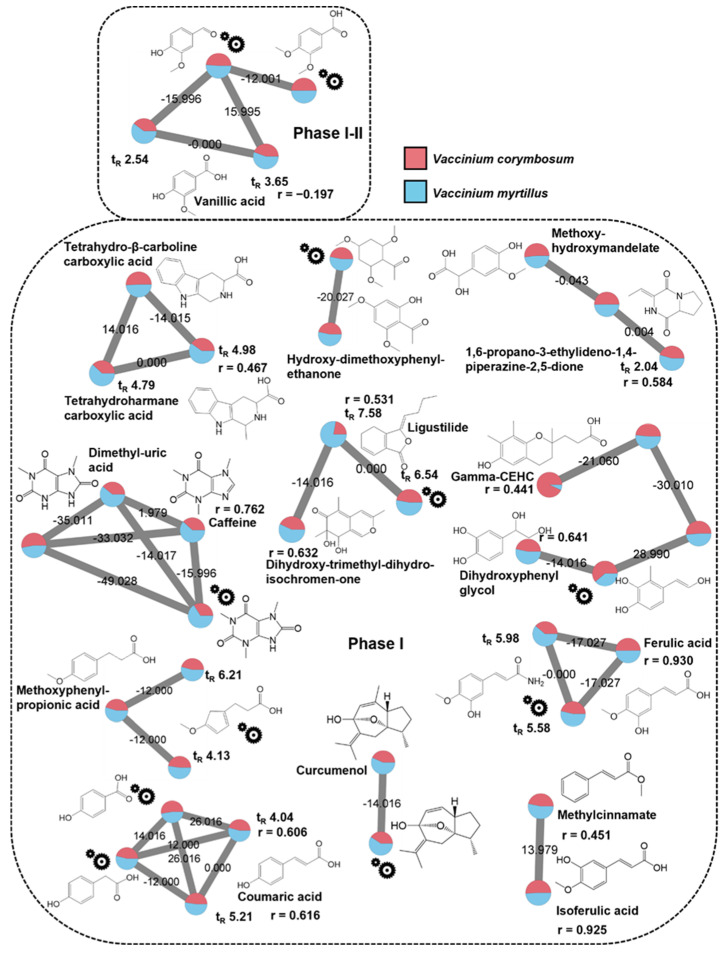
Extracted molecular families of identified metabolites in positive ionization mode listed in Table S2, belonging to the category of (poly)phenolic derivatives and plant endogenous constituents. Dashed boxes group the identified metabolites according to the phase I and II metabolism following PPMC analysis. The “gear” symbols refer to the putative structure identified by manual investigation as reported in Section 2.6 of the main text. Statistically significant Pearson correlation coefficients (r) are reported. Edge labels refer to the mass difference between two nodes.

The other annotated compounds occurred inside molecular families (Table S2), allowing for identifying interesting metabolites. Figure 4 displays the molecular families occurring in the PI dataset with the unknown nodes labelled by “gear” symbols for which were provided hypothesized structures (Figure S7 of the Supplementary Materials). The match with the PI “Nutri-Metabolomics” library identified two nodes as isomers of vanillic acid at different retention times, whereas the remaining nodes were putatively addressed as protocatechuic acid derivatives with high mass accuracy (from −3.26 to −0.06 ppm), by structural elucidation (Figure S7). This molecular family resulted the only one with mixed phase II-phase I contributions. In fact, vanillic acid was characterized by a statistically significant negative r-value, suggesting its direct origin from the supplements intake [35], whereas the two hypothesized protocatechuic acid derivatives exhibited an increasing signal around 6–24 h when their signals were plotted manually, probably originating from microbiota activity [30]. Most identified molecular families were related to phase I metabolism (0.441 < r < 0.930) and, interestingly, several identified and hypothesized node structures can be addressed as metabolite of the native polyphenols occurring in the bilberry and blueberry supplements [30]. Furthermore, derivatives of phloroglucinol carboxylic acid (i.e., hydroxy-dimethoxyphenyl-ethanone), cinnamic acid (i.e., coumaric acid, methylcinnamate, ferulic and isoferulic acid), and mandelic acid (i.e., methoxy-hydroxymandelate) were recognized. A deeper network inspection revealed the occurrence of in-source fragmentations of the conjugation of cinnamic acid derivatives. In detail, at the same t_R_ value of the compound annotated as ferulic acid (t_R_ = 5.14, *m*/*z* = 177.05), the feature at *m*/*z* 252.09 fragmented originating ions at *m*/*z* 177 (methoxycinnamic moiety) and at *m*/*z* 85 (H_4_SO_3_+H^+^ sulfate moiety), suggesting that the annotated compound is a sulfate conjugate. Similarly, vanillic acid (t_R_ = 3.65, *m*/*z* = 169.05) could be addressed as sulfate conjugated, since a feature at t_R_ = 3.7 and *m*/*z* = 261 was characterized by fragments at *m*/*z* = 99 (H_3_SO_4_^+^) and at *m*/*z*=122 (probably benzoic acid). Finally, the compound annotated as isoferulic acid (t_R_ = 4.91, *m*/*z* = 177.05), coeluted with a feature at *m*/*z* = 263, which is probably a derivative of dihydrocaffeic acid sulfate (annotated in NI dataset), thus supporting the sulfated conjugation of isoferulic acid. Three additional interesting spectral families were identified as β-carboline derivatives (i.e., tetrahydroharmane carboxylic acid and tetrahydro-β-carboline carboxylic acid), previously identified in serum samples from this study [18], xanthine pathway metabolites (i.e., dimethyl-uric acid, caffeine), and terpene derivatives (i.e., curcumenol). Regarding xanthine derivatives, even though the identification of uric acid derivatives is in accordance with literature [36], the occurrence of caffeine has never been reported in association with berries consumption and could be attributed to the consumption of caffeine-rich foods before the fasting period foreseen in the study design and/or within the period of pool samples collection [37]. Additionally, caffeine was annotated with Δ = 6.1 ppm by matching with the Massbank mass spectral library, which includes 64 spectra for caffeine acquired in heterogenous instrumental conditions. Thus, caution should be paid on this annotation. Curcumenol and its hypothesized sesquiterpene derivative were reported in this study as well as dihydroxy-trimethyl-isochromenone and ligustilide isomers (however; they were hardly related to the intake of bilberry and blueberry), as well as dihydroxy-trimethyl-isochromenone and ligustilide isomers. Finally, Gamma-CEHC, an endogenous metabolite of vitamin E [38], occurred inside molecular families, exhibiting a significant and positive r-value (0.441), representing a first report in relation to berry consumption.

## 4. Conclusions

This research investigated for the first time the applicability of the FBMN approach in combination with mass spectral libraries relevant to nutrikinetic studies as well as PPMC analysis to boost the structural annotation of postprandial urinary metabolites and to explore their nutrikinetic behavior within a two-arm intervention study on the intake of VM and VC supplements, as a relevant nutrimetabolomics application.

By using the FBMN approach, 24 and 43 metabolites were annotated with high mass accuracy in NI and PI mode, respectively. The comparison with widely used annotation protocols underlined the great potential of the FBMN workflow in providing the basis for an automated exploratory data analysis workflow resulting in a comprehensive and accurate annotation coverage. The proposed workflow offers a wider exploration of the urinary metabolome and allows for a prioritization strategy based on qualitative information. Additionally, the reliability of the presented approach was confirmed by the annotation of biochemically relevant metabolite categories across the three different annotation protocols followed.

The quantitative information introduced by FBMN approach provided an estimation of the impact of the two bilberry intakes on NI and PI datasets. Furthermore, the PPMC analysis of the chromatographic areas of each identified mass feature in relation to the postprandial timepoint proved to be a successful strategy to assess the kinetic shape recognition related to phase I/phase II metabolism of IDs.

It can therefore be concluded that future integration of contextual mass spectral libraries and PPMC analysis within the FBMN environment would be useful for nutrimetabolomics studies, as well as for other omics applications, where boosting annotation rates and streamlining the metabolite selection procedure are key for the data interpretation. Furthermore, it was demonstrated that the automated FBMN approach offers a versatile and scalable alternative to existing approaches that handle untargeted metabolomics profiles of biofluids for biomarker discovery. Finally, our work clearly evidenced the need for curated and contextualized mass spectral libraries that are fundamental for successful metabolite identification and thus biochemical interpretation of metabolomics profiles.

## Figures and Tables

**Table 1:** Number of IDs annotated by Feature-Based Molecular Networking (FBMN) of NI and PI datasets, including the developed “Nutri-Metabolomics” mass spectral libraries, in comparison with the annotation performed with (i) MZmine Library Search using GNPS compatible mass spectral libraries (ALL_GNPS, https://gnps-external.ucsd.edu/gnpslibrary) (accessed on 5 September 2022) and with (ii) the statistical-based approach followed by manual annotation, reported in Section S5 of the Supplementary materials.

Number of IDs	NI	PI
MZmine ^1^	26	49
Statistical-based approach & manual annotation	12	6
FBMN	24	43

^1^ Library search performed at full scan MS level using *m*/*z* and isotope pattern matching.

## Data Availability

Data available in MassIVE accessible repository (MSV000088336). The mass spectral libraries presented in this study are openly available in at https://gnps.ucsd.edu/ProteoSAFe/gnpslibrary.jsp?library=GNPS-NUTRI-METAB-FEM-POS (accessed date 9 November 2021) and https://gnps.ucsd.edu/ProteoSAFe/gnpslibrary.jsp?library=GNPS-NUTRI-METAB-FEM-NEG (accessed date 9 November 2021).

## Supplementary Materials

The following supporting information can be downloaded at: https://www.mdpi.com/article/10.3390/metabo12101005/s1, Supplementary Materials, List of Referece Standards used to build the library, Metadata and Library Information.
