# Supplementary material for: Combining Feature-Based Molecular Networking and Contextual Mass Spectral Libraries to Decipher Nutrimetabolomics Profiles
Source: Metabolites. 2022 Oct 21;12(10):1005. doi: 10.3390/metabo12101005 (PMC9610267; doi:10.3390/metabo12101005)
Supplement: Supplementary file 1 [file metabolites-12-01005-s001.zip › SUPPLEMENTARY MATERIALS.pdf]

Supplementary materials of the Manuscript: "Combining Feature-Based Molecular Networking and contextual mass spectral libraries to decipher nutrimetabolomics profiles.", by L. Renai et al.

Table of contents

S1. Chemicals and reagents 2

S2. Supplement characterization and study design 2

S3. Sample extraction 3

S4. LC-MS/MS analysis 3

S5. Molecular networking analyses, comparative annotation protocols, and assessment of biological significance 4

S6. MS/MS Spectra and schemes for hypothesized structures 14

S1. Chemicals and reagents

Methanol and acetonitrile LC-MS Ultra CHROMASOLV™ and formic acid were purchased from Sigma Aldrich (St. Louis, MO, USA). The ultrapure water was obtained by purifying demineralized water in a Milli-Q system from Millipore (Bedford, MA, USA). The internal standard trans-cinnamic-d5 acid (CIN-d5) was purchased from CDN ISOTOPES Inc. (Pointe-Claire, Quebec, Canada). The surrogate standards L-tryptophan- 2′,4′,5′,6′,7′-d5 (TRY-d5) and N-benzoyl-d5-glycine (hippuric acid-d5, HIP-d5) were obtained from Sigma Aldrich and CDN ISOTOPES Inc., respectively.

S2. Supplement characterization and study design

In order to assess the amount of total soluble polyphenols (TSP) and of total monomeric anthocyanins (TMA) occurring in *Vaccinium Myrtillus* and *Vaccinium Corymbosum* supplements, the following protocols were adopted. About 500 mg aliquots of supplement were homogenized in an ice bath under magnetic stirring with 15 mL of a methanol/water solution 8/2 (v/v) containing 10 mM NaF to inactivate polyphenol oxidase; the mixture was centrifuged at 1800xg for 5 min and the supernatant recovered. This procedure was repeated twice and the extracts combined and analysed for TSP, TMA, and selected phenolic compounds. TSP were spectrophotometrically determined as following described. Extract aliquots (100-200 μL, depending on the TSP concentration in the extract) were mixed with 200 μL of Folin-Ciocalteau reagent. After 3 min, 400 μL of an aqueous solution saturated with sodium carbonate were added and the mixture obtained was made up to 10 mL with ultra-pure water. The solution was dark incubated for 1 h. Afterwards, the absorbance was measured at 740 nm and TSP concentration calculated on the basis of a catechin calibration curve; accordingly, the results were expressed as milligrams of catechin/g of supplement. TMA were determined with the pH differential method using cyanidin-3-glucoside as reference standard. Aliquots of 100-200 μL of extract were diluted in buffer solutions at pH=1 and pH=4.5, so as to obtain a final volume of 10 mL. The absorbance (Abs) of both solutions were measured at 520 and 700 nm and the quantity "ΔAbs" was calculated according to equation 1.

$$\Delta Abs = \left(Abs_{pH=1}^{520\,nm} - Abs_{pH=1}^{700\,nm}\right) - \left(Abs_{pH=4.5}^{520\,nm} - Abs_{pH=4.5}^{700\,nm}\right) \qquad (1)$$

Similarly, "ΔAbs" values were also calculated for different concentrations of cyanidin-3-glucoside reference standard and plotted as a function of corresponding cyanidin-3-glucoside concentrations. The equation best fitting the experimental data was calculated by the least square regression method, thus obtaining a linear calibration curve, which was used for measuring TMA in the extracts. The results were expressed as milligrams of cyanidin-3-glucoside per g of supplement.

Table S1 – Total soluble polyphenol (TSP) and total monomeric anthocyanin (TMA) concentrations of *V. myrtillus* and *V. corymbosum* supplements administered to volunteers during the study.

	V. myrtillus supplement	*V. corymbosum* supplement
TSP (mg CAT/g supplement dry weight)	38.8 ± 2.1	9.3 ± 1.0
TMA (mg CYA-3-GLU/g supplement dry weight)	30.1 ± 2.8	6.4 ± 0.9

The randomized, single-blinded two-arms intervention study involved twenty healthy voluntary subjects (11 males and 9 females aged between 25 and 60 years). All the subjects convened early in the morning after 10 h of fasting and were randomly divided in two groups according to an electronic randomisation key. Volunteers were asked to avoid fruit and vegetable consumption from fasting to 48 hours after supplement intake. A single dose of 25 g of VM supplement mixed with 500 mL of water was orally administered to the first group, whereas the same amount of VC berry supplement mixed with 500 mL of water was orally administered to the second group.

S3. Sample extraction

After the collection, samples were divided in aliquots of 500 µL, frozen at -80ºC and stored until extraction and LC-MS/MS analysis were performed.100 µL of urine were placed into the Millipore 96-well plate (Waters, Milford, MA, USA) and 100 µL of surrogate standards in methanol (25 µg/mL TRI-d5 and HIP-d5) were added. Then, a cap mat was applied on the well plate and the system was mixed for 5 minutes. After mixing, the well plate and the collection plate were placed on the positive pressure processor (Positive Pressure-96 Processor, Waters) and the filtration was performed setting the flow for 60 psi for 5 minutes. Then, 300 µL of internal standard (0.83 µg/mL CIN-d5) were added to each position of the collection plate and the system was mixed for 5 minutes. The resulting filtrated and diluted urine samples were transferred in labelled brown vials for LC-MS analysis. For testing the urine filtration procedure, a quality control (QC) sample consisting of a pool of the same volume of all urine samples was prepared. The same extraction protocol was performed on QC aliquots randomly placed in the well plate. The QC extracts together with the extraction blanks were injected before the LC-MS analysis of individual samples.

S4. LC-MS/MS analysis

The chromatographic analysis was performed on a Kinetex C18 column and a guard column containing the same stationary phase. Column temperature was set at 40°C. Water (eluent A) and acetonitrile (eluent B), both with 0.1 % formic acid, were used as mobile phases. For chromatographic analysis of urine sample, the following gradient was adopted: 0-1 min isocratic 5% B, 1-7 min linear gradient 5-45% B, 7-8.5 min linear gradient 45-80% B, 8.5-10.5 min isocratic 80% B, 10.5 -11 min linear gradient 80-5% B, 11-12 min 5% B. Flow rate was 350 µL/min and the injection volume was

5 μL. The ESI conditions in positive (and negative) mode were: spray voltage 5.0 kV (-3.5 kV), heated capillary temperature 320ºC, capillary voltage 35 V (-35 V) and tube lens 110 V (-110 V). In the LTQ component of the instrument, nitrogen was used as both the sheath gas (35 U) and auxiliary gas (5 U), and helium was used as the damping gas. All measurements were done using the automatic gain control of the LTQ to adjust the number of ions entering the trap. Mass calibration was performed with every sequence run just prior to starting the batch by using flow injection of the manufacturer's calibration standards mixture allowing for mass accuracies lower than 5 ppm in external calibration mode. Data-dependent acquisition was performed to obtain both full scan (mass range from 100 to 1000 Da) and MS/MS spectra with a resolving power for MS/MS scans of 7500. Product ions were generated in the LTQ trap at collision energy 35 eV (for both CID and HCD) using an isolation width of 2 Da.

S5. Molecular networking analyses, comparative annotation protocols, and assessment of biological significance

In detail, precursor ion mass tolerance (PIMT) and fragment ion mass tolerance (FIMT) were investigated in the range of 0.01-1 Da, since they can act as inclusion/exclusion parameters for the clustering of the occurring features and they need to match the dataset well. The number of minimum matched fragment ions to create a consensus was investigated from 2 to 7, as it is also responsible for the creation of a common consensus spectrum. Networking and library cosine scores are parameters related to the similarity degree between consensus and library spectra, used for network creation and annotation, respectively; thus, they were investigated in the range of 0.2-0.7, to test the accuracy of networking and library matching. Finally, the minimum number of library shared peaks was studied from 3 to 6 to reduce the number of incorrect annotations. A comparative analysis among these settings was performed by looking at number of IDs, molecular families, nodes, and edges.

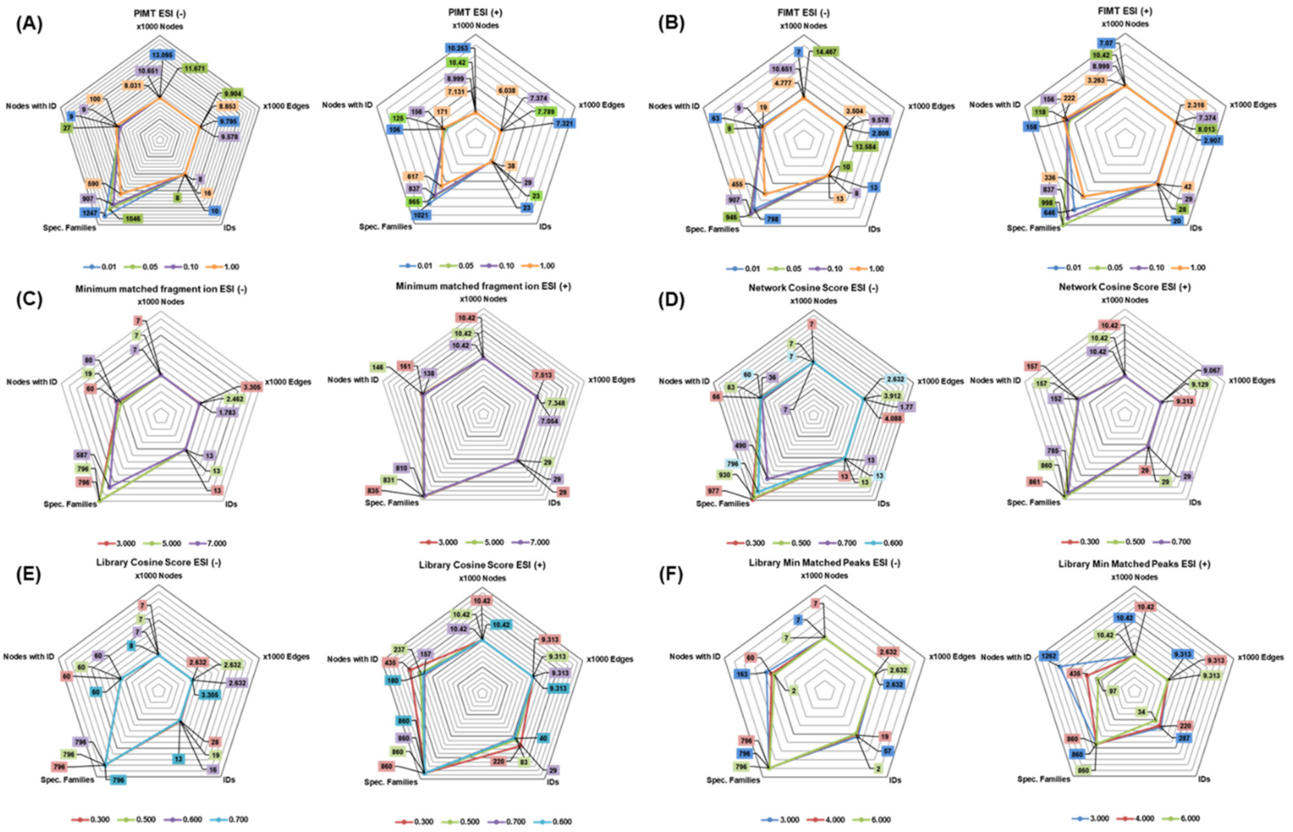

Figure S1 – Radar graphs of the molecular networking outputs for the selection of basic and advanced options (see paragraph 3.1 of the main text) to be used in Feature-Based Molecular Networking workflow for both negative ionisation and positive ionisation datasets. (A) Precursor ion mass tolerance (PIMT). (B) Fragment ion mass tolerance (FIMT). (C) Minimum matched fragment ions. (D) Networking cosine scores. (E) Library cosine scores. (F) Minimum number of library shared peaks.

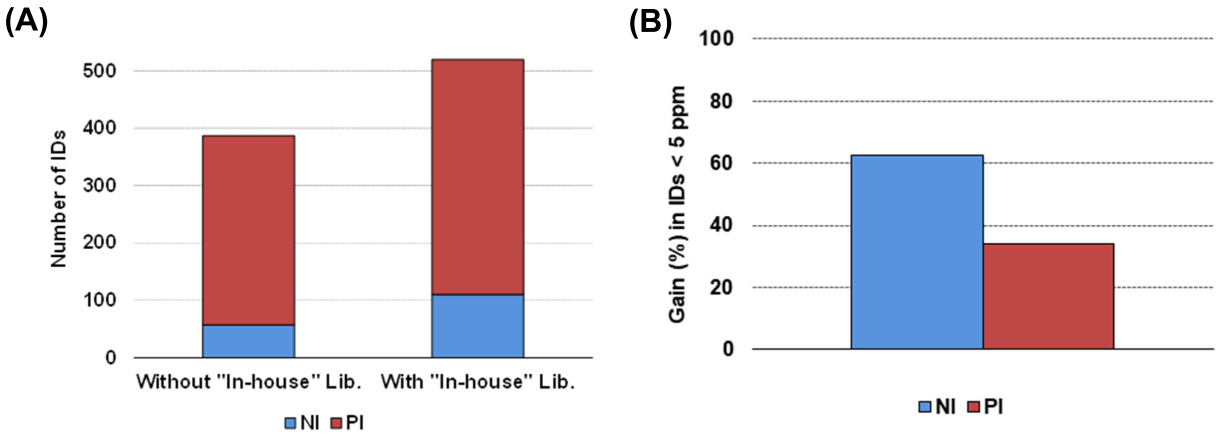

Figure S2 – (A) Number of identified nodes (IDs found by Molecular Networking) from both negative ionisation and positive ionisation datasets before and after the inclusion of the "Nutri-Metabolomics" library. (B) Percentage of gain (Δ%) in IDs found within 5 ppm mass accuracy including the "Nutri-Metabolomics" library versus without including the contextual library.

Table S2 – List of the metabolites (ID) annotated by Feature-Based Molecular Networking of NI and PI datasets, including the developed "Nutri-Metabolomics" libraries (Nut.-Met.) that contains 319 and 339 metabolites in negative and positive ionisation modes, respectively. Retention time (t_R, min), mass of precursor ion (MS, Da), type of adduct, product ions (MS/MS, Da) of query spectra and relative base peak in bold, mass error of precursor ion vs. theoretical mass (Δ, ppm), type of library used for annotation, library cosine score, kind of occurrence in the network (NW), i.e. as singleton (S) or molecular family (MF), and PPMC r-values of the postprandial kinetics. The abbreviations n.a. and n.s. mean library match not available and not significant PPMC, respectively.

#	ID	t_R	MS	Adduct	MS/MS	Δ	Library	Score	NW	r-value
	NI dataset									
**1**	Hydroxyphenyl propionic acid	4.95	165.056	M-H	**121.066**; 121.028	0.5	Nut.-Met.	0.78	MF	-0.415
**2**	Dihydroxyphenyl propanoic acid glucuronide	3.10	357.083	M-H	341.272; 175.0248; **113.0245**	2.9	Nut.-Met.	0.74	MF	-0.302
**3**	Methoxyindole acetic acid	6.40	204.067	M-H	**160.077**; 130.066; 117.071	1.6	Nut.-Met.	0.82	MF	n.s.
**4**	Hydroxy-methoxycinnamic acid	4.80	193.051	M-H	**178.027**; 149.061; 134.037	0.2	Nut.-Met.	0.82	MF	n.s.
**5**	Isovanillic acid	3.71	167.035	M-H	152.011; **123.045**; 108.022	0.6	Nut.-Met.	0.77	MF	n.s.
**6**	α-Hydroxyhippuric acid	4.73	194.046	M-H	**150.056**	1.0	Nut.-Met.	0.90	MF	n.s.
**7**	Azelaic acid	6.54	187.098	M-H	**125.0972**	1.5	Nut.-Met.	0.97	S	0.570
**8**	Dihydrocaffeic acid sulphate	4.44	261.007	M-H	**181.050**	1.8	Nut.-Met.	0.91	MF	0.526
**9**	Dihydroferulic acid sulphate	4.66	275.023	M-H	**195.066**	2.6	Nut.-Met.	0.92	MF	0.700
**10**	Furoylglycine I	2.61	168.03	M-H	**124.040**; 100.004; 67.019	2.2	Nut.-Met.	0.89	MF	0.429
**11**	Furoylglycine II	2.91	168.03	M-H	**124.040**; 100.004; 67.019	2.2	Nut.-Met.	0.89	MF	0.940
**12**	Galacturonic acid	6.38	193.035	M-H	199.888; 175.025; 131.035; **113.025**	2.5	Nut.-Met.	0.55	S	-0.266
**13**	Glutamine	4.81	145.062	M-H	128.036; **127.052**; 109.041	1.4	Nut.-Met.	0.56	S	n.s.
**14**	Hippuric acid	4.74	178.051	M-H	**134.0609**	0.9	Nut.-Met.	0.97	MF	0.348
**15**	Homovanillic acid sulphate	4.00	261.007	M-H	217.017; **181.050**; 137.061	1.8	Nut.-Met.	0.86	MF	0.526
**16**	Indoxyl sulphate	4.75	212.002	M-H	132.045; **80.965**; 79.958	0.9	Nut.-Met.	0.94	MF	0.567
**17**	Isoferulic acid glucuronide	4.92	369.083	M-H	193.050; 175.024; **113.024**; 95.0139	1.7	Nut.-Met.	0.87	MF	0.757
**18**	Dihydrocaffeic acid glucuronide I	3.90	357.082	M-H	**313.093**; 175.025; 113.025	1.7	Nut.-Met.	0.60	MF	n.s.
**19**	Dihydrocaffeic acid glucuronide II	4.10	357.082	M-H	**313.093**; 175.025; 113.025	1.7	Nut.-Met.	0.60	MF	0.395
**20**	Ethoxy-oxobutenoic acid	2.87	143.035	M-H	99.045; 81.035; **71.014**	0.7	Massbank	0.83	S	0.880
**21**	Enterolactone	6.79	297.113	M-H	**253.123**; 189.0552; 165.056; 121.066	0.3	Massbank	0.93	MF	n.s.
**22**	Dihydroxybenzene sulfonate	3.73	188.986	M-H	**109.030**; 80.965; 79.958	0.6	Massbank	0.60	MF	n.s.
**23**	Methoxyabscisic acid glucuronide	7.09	453.175	M-H	435.166; **277.144**; 233.155; 175.025	2.6	n.a.	n.a.	MF	n.s.
**24**	p-Cresol sulphate	5.64	187.009	M-H	187.007; 107.057; **79.958**	1.1	A.Walker	0.92	MF	-0.536

Table S2 – Continued

#	ID	t_R	MS	Adduct	MS/MS	Δ	Library	Score	NW	r-value
	PI dataset									
**1**	Tetrahydroharmane carboxylic acid	4.79	231.113	M+H	**214.086**; 188.070; 158.096	0.9	Nut.-Met.	0.74	MF	0.467
**2**	Hydroxy-dimethoxyphenyl-ethanone	5.60	197.080	M+H	169.086; **151.075**; 133.101	5.0	BMDMS-NP	0.30	MF	n.s.
**3**	1,6-propano-ethylideno-1,4-piperazine-2,5-dione	2.04	181.096	M+H	153.102; 149.023; **125.071**	4.5	Liaw-Yang	0.80	MF	0.584
**4**	Dimethyl-uric acid	3.51	197.067	M+H	182.043; 158.056; **140.045**	5.1	Dorrestein	0.87	MF	n.s.
**5**	β-Glucopyranosyl-tryptophan	1.90	367.150	M+H	349.139; 331.129: **247.108**; 229.097	0.8	C. Maier	0.40	S	n.s.
**6**	Dihydroxycinnamic-trihydroxycyclohexane carboxylic acid	4.54	355.102	M+H	341.216; **285.010**; 266.999; 163.039	4.7	MoNA	0.35	S	0.373
**7**	Tetrahydro-β-carboline carboxylic acid	4.52	217.097	M+H	173.107;171.091; **144.081**	0.2	Nut.-Met.	0.97	MF	n.s.
**8**	Dihydroxyphenylglycol	6.06	153.054	M-H2O+H	135.116; **107.049**	2.0	Dorrestein	0.60	MF	0.641
**9**	Methoxyphenyl-propionic acid	8.13	163.075	M-H2O+H	135.080; 121.1006; **107.085**	2.4	Nut.-Met.	0.68	MF	n.s.
**10**	Methoxy-hydroxymandelate	2.03	181.049	M-H2O+H	153.055; **149.023**; 125.071	5.0	Dorrestein	0.64	MF	n.s.
**11**	Ferulic acid	5.14	177.054	M-H2O+H	**145.028**	3.0	Nut.-Met.	0.98	MF	0.930
**12**	Vanillic acid	3.65	169.050	M+H	**125.059**; 93.033	0.5	Nut.-Met.	0.96	MF	-0.197
**13**	Dihydroxy-trimethyl-isochromenone	7.37	223.096	M+H	205.085; **181.086**; 163.075	2.3	MoNA	0.40	S	0.469
**14**	Absicic Acid	7.38	247.133	M-H2O	**229.122**; 211.111; 201.127; 187.111	0.4	Nut.-Met.	0.88	S	n.s.
**15**	Alpha-CEHC	6.96	261.148	M-H2O+H	**243.138**; 165.091	1.6	Nut.-Met.	0.44	S	n.s.
**16**	Azelaic acid	6.55	171.101	M-H2O+H	153.090; **125.096**	2.2	Nut.-Met.	0.57	S	0.640
**17**	Cinnamic acid	4.86	149.060	M+H	**131.049**	4.0	BMDMS-NP	0.46	S	n.s.
**18**	Curcumenol	6.86	235.169	M+H	**217.158**; 189.163; 137.0592; 111.0800	0.4	MoNA	0.40	MF	n.s.
**19**	Dihydro-deoxy-lactucin	6.41	263.129	M+H	**245.117**; 217.122; 188.091	6.5	HTSimonsen	0.61	S	0.878
**20**	Ethylindole carboxylic acid	8.01	190.086	M+H	172.075; **144.080**; 118.065	0.2	Nut.-Met.	0.65	S	0.958
**21**	Folinic acid	3.78	474.173	M+H	456.162; **345.130**; 327.119	1.5	Nut.-Met.	0.60	S	0.546
**22**	Formylkynurenine	2.14	237.087	M+H	**220.061**; 202.050; 192.066	3.8	Quinn	0.40	S	-0.645
**23**	Furaneol	4.03	129.055	M+H	111.044; **101.059**	2.5	Nut.-Met.	0.65	S	n.s.
**24**	Furoylglycine	2.68	152.034	M-H2O+H	**124.039**; 95.013	3.5	Nut.-Met.	0.98	S	0.967
**25**	Gamma-CEHC	6.68	247.132	M-H2O	**229.122**; 203.106; 151.075	2.7	Nut.-Met.	0.65	MF	0.441
**26**	Indole acetic acid	5.33	176.071	M+H	145.028; **131.068**	2.3	M. Ernst	0.53	S	0.512
**27**	Isoferulic acid	4.91	177.054	M-H2O+H	163.039; **145.028**; 117.033	3.0	Nut.-Met.	0.92	MF	0.925
**28**	Methylcinnamate	4.96	163.075	M+H	**131.075**; 103.054	1.2	Massbank	0.86	MF	0.451
**29**	Caffeine	4.63	195.088	M+H	**138.066**	6.1	Massbank	0.97	MF	0.762
**30**	Trihydroxybutyrophenone	6.37	197.081	M+H	169.086; **151.075**; 137.059	1.5	Massbank	0.59	S	0.496
**31**	Hesperetin	6.70	303.086	M+H	285.186; **177.054**; 153.018	0.7	Massbank	0.83	S	n.s.

Table S2 – Continued

#	ID	t_R	MS	Adduct	MS/MS	Δ	Library	Score	NW	r-value
**32**	Dihydroresveratrol	6.19	231.102	M+H	**213.148**; 203.1062; 137.059; 121.064	3.5	Massbank	0.82	S	0.528
**33**	m-Coumaric acid	5.21	147.044	M-H2O+H	**119.049**; 105.069	1.3	Nut.-Met.	0.74	MF	n.s.
**34**	Dihydroxy-trimethyl-dihydro-isochromen-one	4.29	223.096	M+H	**205.086**;163.075	4.0	Dorrestein	0.62	MF	0.632
**35**	Nerol	8.26	137.132	M-H2O+H	**95.085**; 81.070	2.7	Nut.-Met.	0.45	S	-0.164
**36**	Coumaric acid I	4.04	147.044	M-H2O+H	130.050; **119.049**	2.0	Nut.-Met.	0.89	MF	0.606
**37**	Coumaric acid II	5.21	147.044	M-H2O+H	130.050; **119.049**	2.0	Nut.-Met.	0.89	MF	0.616
**38**	Sebacic acid	7.33	185.117	M-H2O+H	**167.106**; 121.101	2.9	Nut.-Met.	0.56	S	-0.214
**39**	Ketodeoxycholic acid	8.14	373.274	M+H-H2O	**355.262**; 337.252	1.9	R. Knight	0.40	S	n.s.
**40**	Keto-octadecadienoic acid	8.05	295.226	M+H	**277.216**; 249.221	0.9	R. Knight	0.40	S	0.387
**41**	Hydroxy-methoxybenzophenone	7.17	229.087	M+H	**151.039**;105.033	5.3	W. H.Gerwick	0.80	S	-0.284
**42**	Enterolactone	6.78	281.117	M+H-H2O	**263.106**; 149.060; 133.065	2.8	R. Knight	0.82	S	0.886
**43**	Ligustilide	7.59	191.107	M+H	163.075; **145.101**; 117.070	2.6	J. L. Wolfender	0.56	MF	0.531

The MZmine Library Search was performed on the same feature list used for the FBMN workflow, using the GNPS library format (ALL_GNPS) available at https://gnps-external.ucsd.edu/gnpslibrary. All query scans contained in the feature list were matched against the imported spectral library at the Full Scan MS level, since a complete "mass list" in MZmine is needed to perform Library Search, which was not possible to obtain for all the MS/MS scans. The parameters for Library Search were set as follows for both NI and PI datasets: precursor m/z tolerance at 5 ppm, minimum ion intensity at 7.0×10^3, spectral m/z tolerance at 10 ppm, and minimum matched signals at 3, selecting as similarity score the weighted dot-product cosine.

Biomarkers of food intake in postprandial responses were selected adopting the following conventional protocol. For each feature (i.e., a m/z at a given retention time), the baseline sample was subtracted from the intensities of the other time points, and negative values were replaced by zero. Features that in at least 2 consecutive points exhibited the 25th percentile of one group higher than the 75th percentile of the other group, were included in the discrimination process based on comparison of area under the curve (AUC). The AUC of each selected feature was calculated between 0 min and 48h time points, using the pracma R package, whereas differences of AUCs among diets were tested using the Wilcoxon-Mann Whitney test and the obtained P-values were adjusted using the Benjamini Hochberg (BH) method. Adjusted P-values < 0.05 were considered statistically significant. The annotation of the significant features was performed using the workflow described below. (i) Comparison of the exact mass of the experimental precursor ion with the pseudo-molecular ions proposed by the MzCloud, Humane Metabolome Database, Mass- Bank of North America, and Kyoto Encyclopedia of Genes and Genomes libraries, selecting a mass accuracy (Δ) $\leq$ 5 ppm as tolerance threshold. (ii) Export of the isotopic profile of pseudo-molecular ions selected within libraries at step (i) and subsequent comparison with the isotopic profile of the experimental precursor ions, selecting features providing an isotope ratio percentage difference of 20% as tolerance threshold. (iii) Structural elucidation of features of interest through the evaluation of MS/MS spectra obtained with DDA mass method in comparison with matched library mass spectra. (iv) Feature annotation performed according to golden standards for metabolomics.

Valerolactone and valeric acid derivatives were annotated as follow. In detail, features at m/z 383.10 (dihydroxyphenyl-γ-valerolactone glucuronide II) and 287.02 (dihydroxyphenyl-γ-valerolactone sulfate) occurred inside the same molecular family inside NI dataset, sharing the common MS/MS bas peak at m/z 207.06 (Figure S2A and S2B of the Supplementary materials) corresponding to the loss of glucuronide and sulfate moieties for the former and the latter precursor ions, respectively. Inside PI dataset, only the feature at m/z 289.04 (dihydroxyphenyl-γ-valerolactone sulfate) gave rise

to a singleton in the network, confirmed by the fragmentation pattern (Figure S2C) given for this node in comparison with Table S3.

Table S3 – List of metabolites tentatively identified by conventional untargeted protocol in urine samples after acute ingestion of VM and VC supplements. For each statistically significant feature retention time (t_R), precursor ion exact mass (Da) and charge state in squared brackets, product ions (MS/MS, Da), the tentative identification and the postprandial statistical significance are reported.

t_R	Precursor ion	MS/MS	Tentative identification	*P*-Value & diet
0.96	128.0191 [M+H]+	MS^2 128: 110.0085 (100); MS^3 128-110: 82.0134 (100).	Hydroxy-furoic acid	8.55E-03, VC
1.42	369.0514 [M+H]+	MS^2 369: 193.0968 (100), 175.0870 (15).	Dihydroxytryptamine/oxoamide glucuronide	1.01E-02, VM
2.24	220.1176 [M+H]+	MS^2 220: 202.1066 (100), 184.0962 (40), 174.1117(10), 90.0544 (40).	Pantothenic acid	2.01E-02, VM
3.07	194.0451 [M+H]+<br>389.0967 [2M-H]-	MS^2 194: 150.0554 (100), 194.0447 (60), 93.0344(17), 148.0399 (12).<br>MS^2 389: 194.0447 (100), 150.0555 (12).	Hydroxyhippuric acid	5.24E-03, VM
3.33	373.0769 [M-H]-	MS^2 373: 197.0452 (100), 182.0212 (15), 175.0247(40), 113.0243 (30).	Syringic acid glucuronide I	5.02E-04, VC
3.54	505.1190 [M-H]-	MS^2 505: 343.0662 (80), 329.0871(100), 191.0346(60), 167.0348(60); MS^3 505-329: 123.050(100).	Vanilic acid glucoside-glucuronide	2.60E-03, VM
3.95	373.0769 [M-H]-	MS^2 373: 197.0452 (100), 182.0217(10), 175.0244 (30), 113.0244 (35).	Syringic acid glucuronide II	2.60E-03, VC
4.22	307.0490 [M+H]+	MS^2 307: 289.0380 (100), 173.0569 (40); MS^3 307-289: 229.0160 (100), 209.0803 (70), 191.0696 (60)	Dihydroxyphenyl-valeric acid sulfate	8.55E-03, VM
4.27	449.1080 [M+H]+	MS^2 449: 287.0543 (100), 317.0655 (30), 143.0432 (5).	Cyanidin-hexoside	2.00E-03, VM
4.54	339.07139 [M-H]-	MS^2 339: 175.0248 (100), 163.0394 (40).	Hydroxycoumarin glucuronide	1.56E-02, VM
4.60	385.1120 [M+H]+	MS^2 209: 191.0697 (100), 149.0593 (44), 163.0755 (5), 123.0442 (4).	Dihydroxyphenyl-γ-valerolactone glucuronide I	2.93E-03, VM
4.80	383.0975 [M-H]-	MS^2 383: 207.0658 (100), 175.0244 (40), 113.0243 (45), 163.0767 (20).	Dihydroxyphenyl-γ-valerolactone glucuronide II	3.96E-03, VM
4.97	289.0373 [M+H]+<br>287.0223 [M-H]-	MS^2 289: 271.0267 (35), 229.0163 (100), 209.0805(50), 191.0701(55), 149.0596(30), 131.0489(60), 143.0316(15)<br>MS^2 287: 207.0622 (100), 163.0762 (3); MS^3 287-207: 163.0760(100), 122.0376 (20), 109.0292 (12).	Dihydroxyphenyl-γ-valerolactone sulfate	2.93E-03, VM
5.16	343.0663 [M-H]-	MS^2 163: 175.0246 (100), 167.0349 (20), 113.0245 (30).	Vanillic acid glucuronide	1.47E-02, VM
5.18	357.0819 [M-H]-	MS^2 357: 181.0502 (100), 137.0611 (40).	Dihydrocaffeic acid glucuronide	7.95E-03, VM
5.25	383.0434 [M-H]-	MS^2 383: 303.0858 (100), 216.9803 (59), 137.0239 (44), 245.0123 (15), 259.0965 (7).	(Epi)catechin-methyl sulfate	1.22E-02, VM
5.33	455.15487 [M-H]-	MS^2 455: 279.1232 (100), 217.1228 (30), 143.1070 (15), 175.0241 (6).	Hydroxy-abscisic acid glucuronide	1.32E-03, VC
6.11	333.0607[M-H]-	MS^2 333: 165.0191 (100), 183.0296(22), 137.0244(20), 289.0709 (19).	Methyl-dihydromyricetin	2.60E-03, VM
		Unknowns		
3.59	208.1138 [M -H] -<br>417.2344 [2M -H] -	MS^2 208: 164.1240 (100), 120.0756(30), 121.0821(28), 146.0914 (10)	Unknown metabolite	6.78E -03, VC
5.11	507.2072 [M -H] -	MS^2 507: 489.1974 (100), 471.1865(20), 447.1870 (60), 189.1824 (15), 175.0249(30), 173.0457(44), 157.0142(30)	Unknown glucuronide metabolite	5.02E -04, VC
5.72	587.1966[M -H] -	MS^2 587: 411.1657 (100)	Unknown glucuronide metabolite	2.10E -03, VM
6.55	423.1287 [M-H]-	MS^2 423: 339.0714 (100), 175.0244 (30)	Unknown glucuronide metabolite	4.55E-03, VM
7.07	327.0537 [M-H]-	MS^2 327: 247.0972(100), 242.9963(50), 163.0398(55), 119.0501(10)	Unknown sulfate metabolite	1.06E-02, VM
8.97	477.2483[M-H]-	MS^2 477: 459.2380 (80), 355.2281(40), 301.2165(100), 175.0244(10)	Unknown glucuronide metabolite	4.98E-03, VM

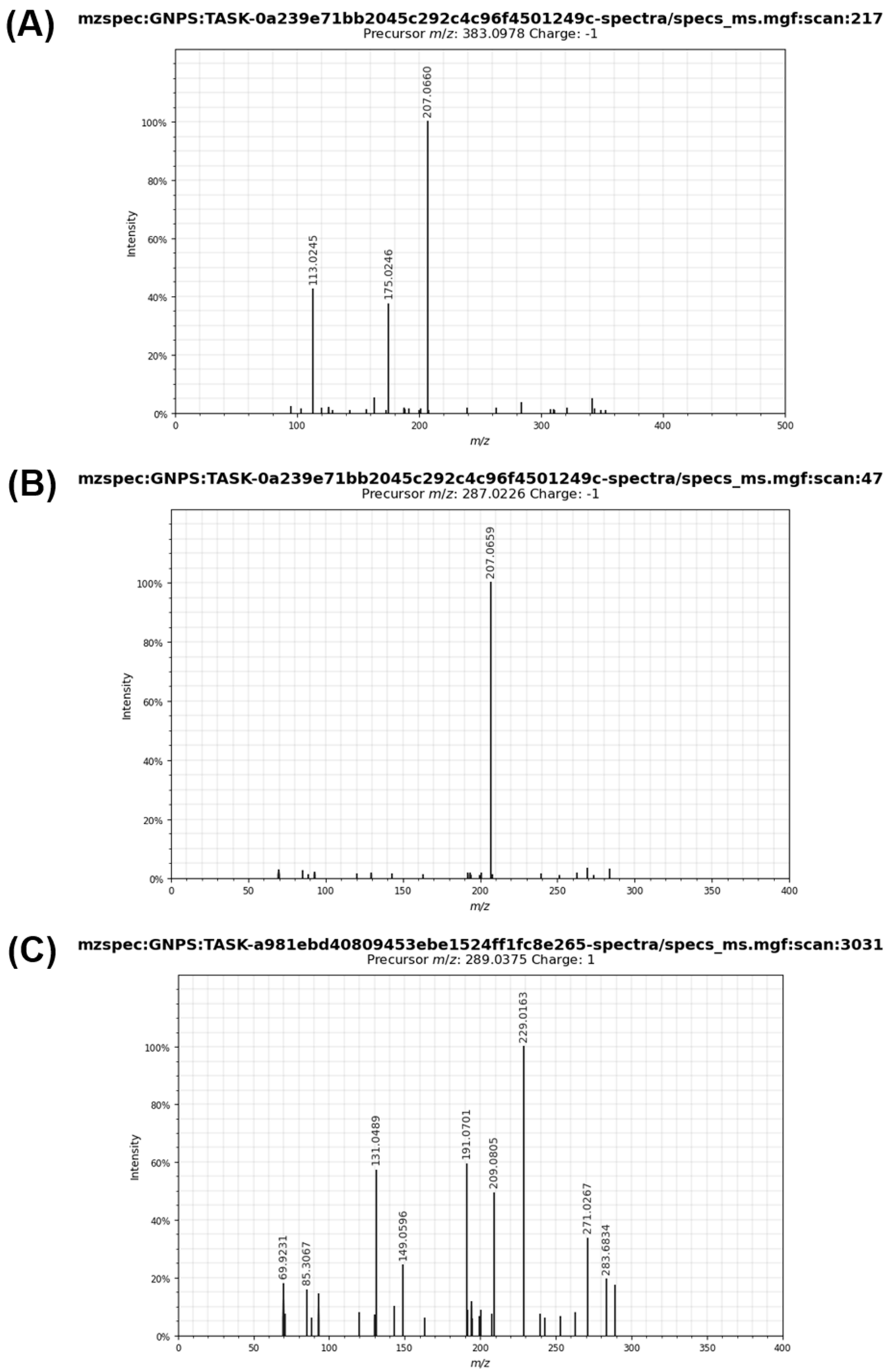

Figure S3 – MS/MS spectra of the putatively annotated valerolactone derivatives reported in Table S2 and occurring inside NI and PI FBMN. (A) Dihydroxyphenyl-γ-valerolactone glucuronide II, *m/z* 383.10 Da. (B) Dihydroxyphenyl-γ-valerolactone sulfate, *m/z* 287.02 Da. (C) Dihydroxyphenyl-γ-valerolactone sulfate, *m/z* 289.04 Da.

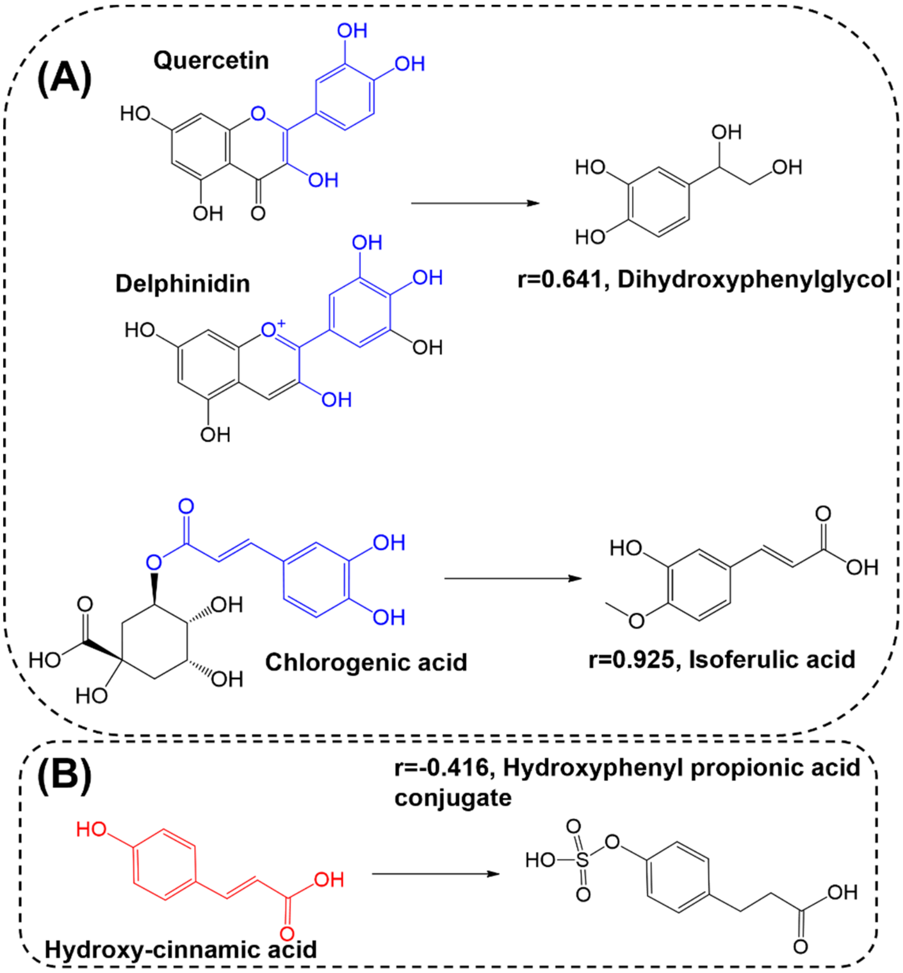

Figure S4 – Schematic examples of structural modification of native phenolics involved in the (A) phase I and (B) phase II metabolism, in relation with the significant PPMC coefficient found for the reported annotated metabolites.

S6. MS/MS Spectra and schemes for hypothesized structures

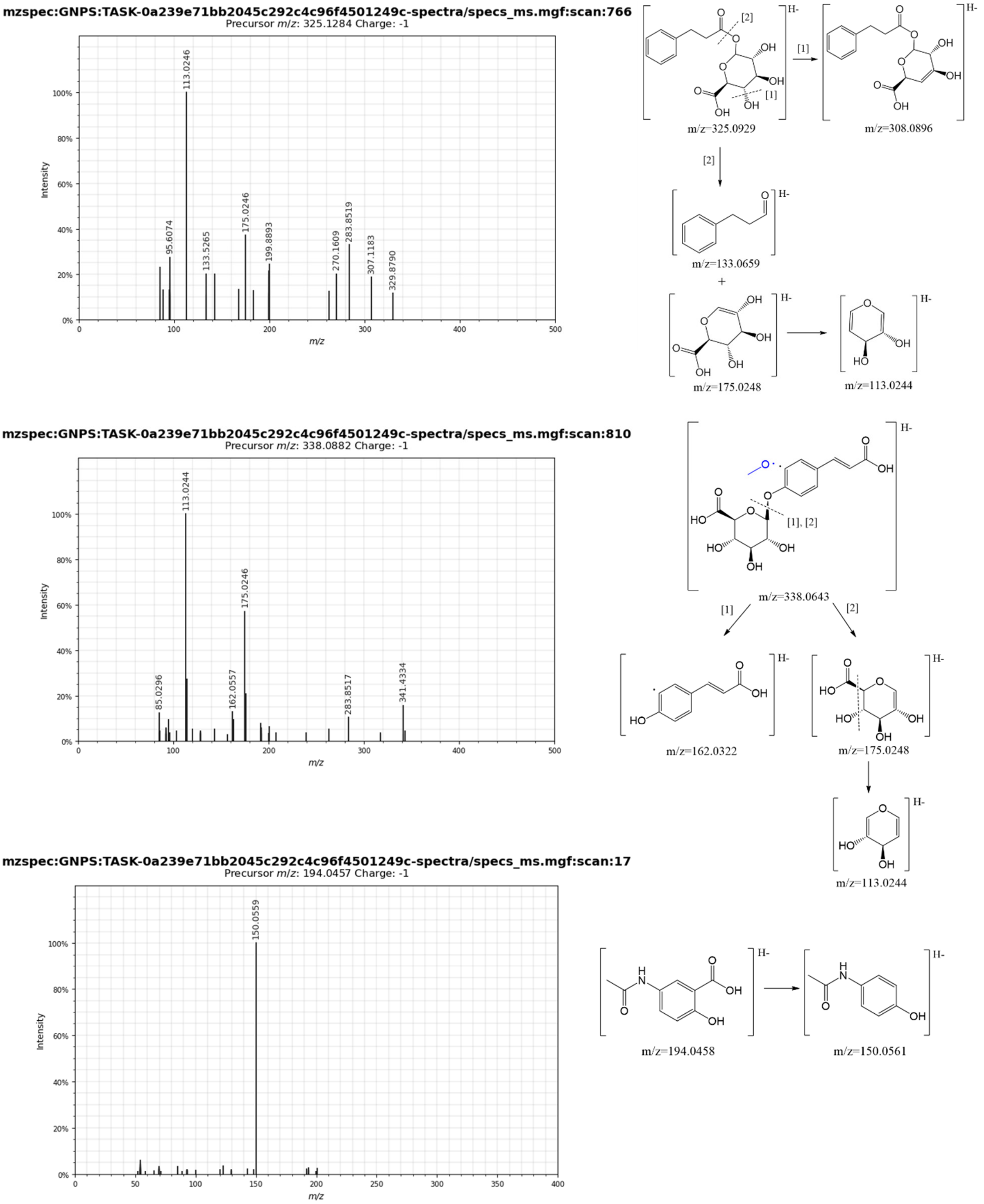

Figure S5 – MS/MS spectra and hypothesized fragmentation scheme of putative metabolites reconstructed from network analysis of Figure 3 of the main text. In-source fragmentations are highlighted whether occurred.

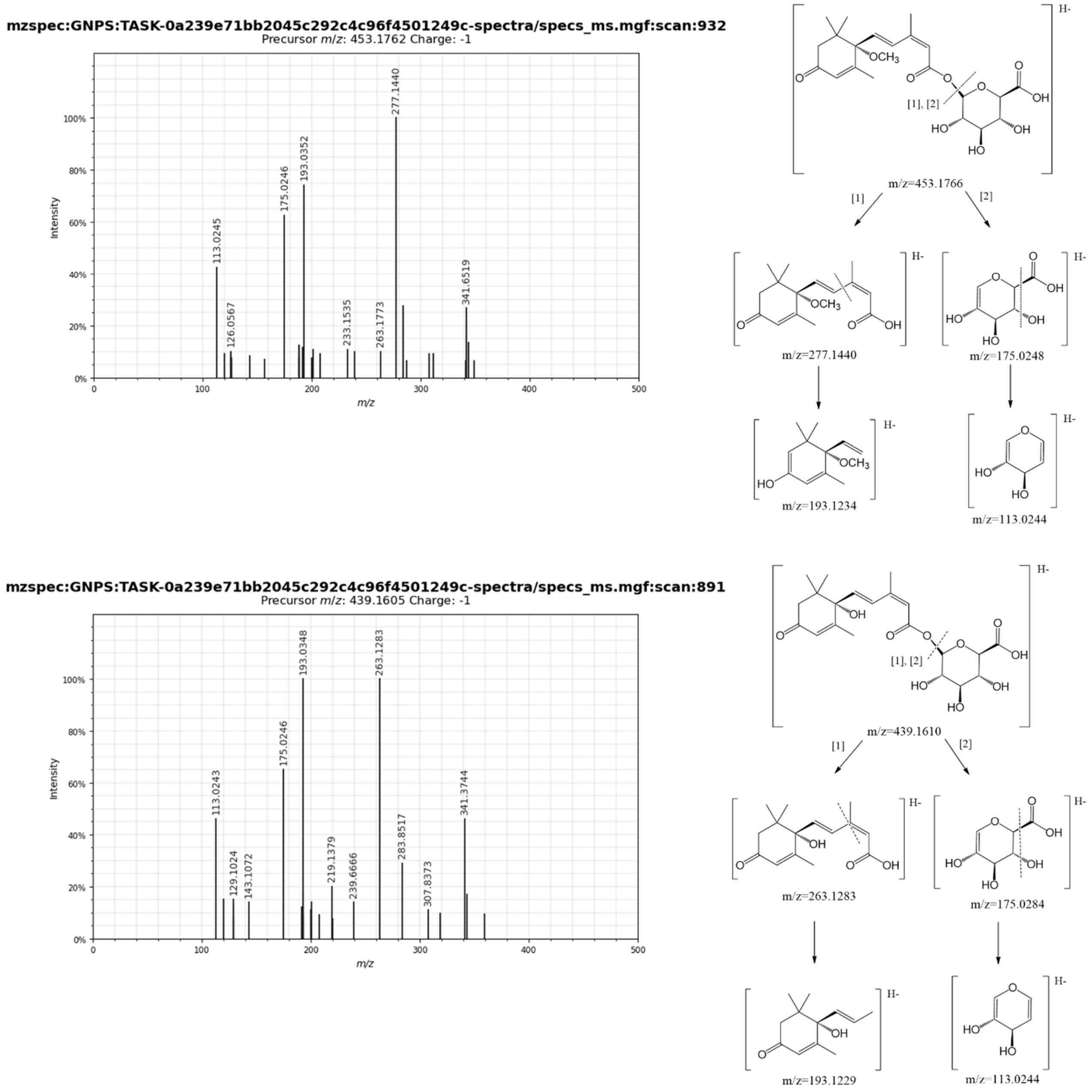

Figure S6 – MS/MS spectra and hypothesized fragmentation scheme of putative metabolites methoxyabscisic acid glucuronide (top) and abscisic acid glucuronide (bottom), reconstructed from network analysis of Figure 3 of the main text.

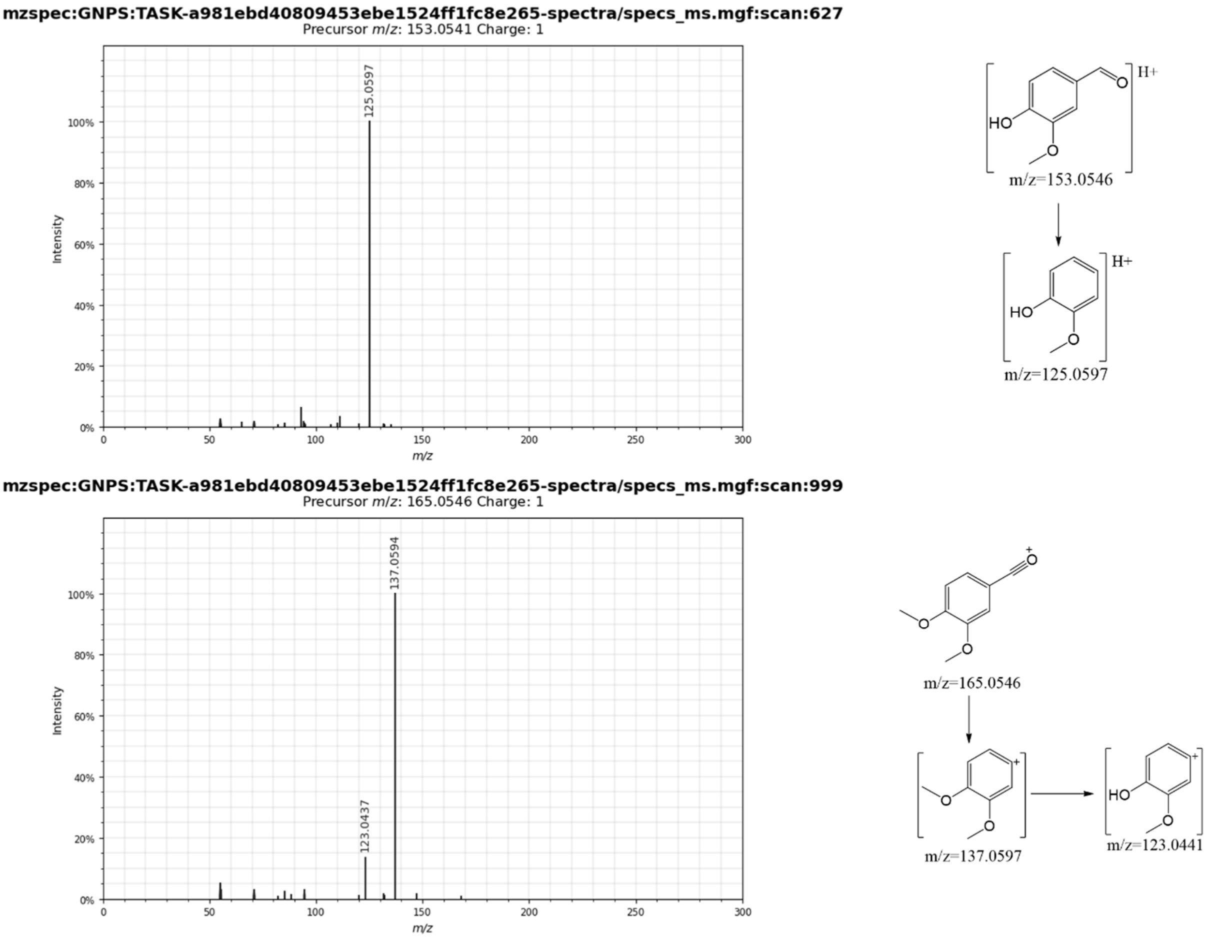

Figure S7 – MS/MS spectra and hypothesized fragmentation scheme of putative metabolites reconstructed from network analysis of Figure 4 of the main text.

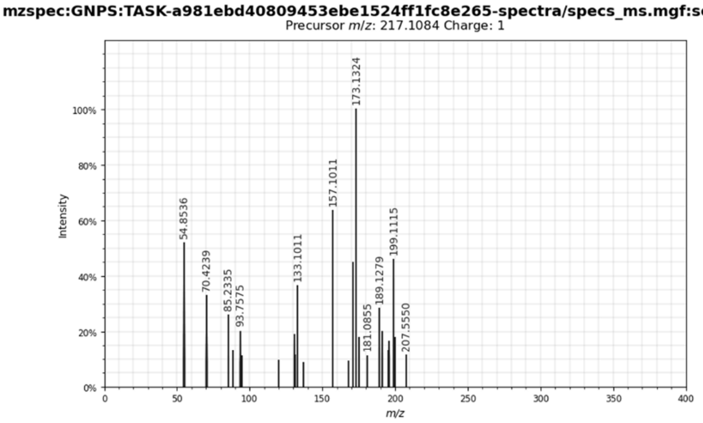

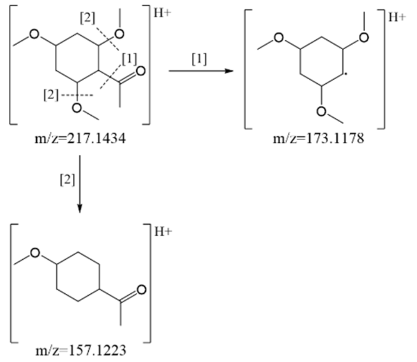

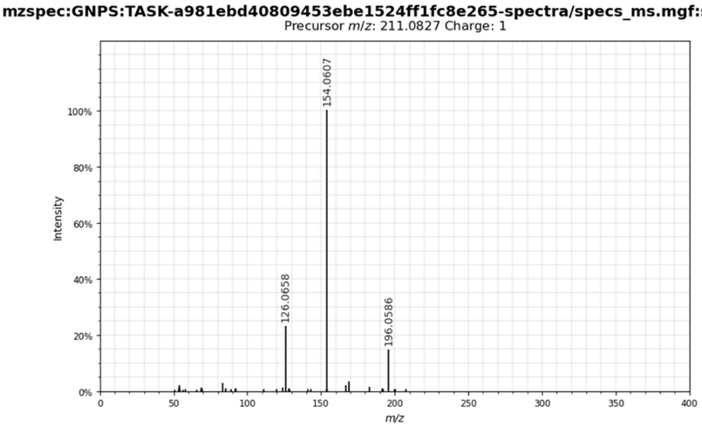

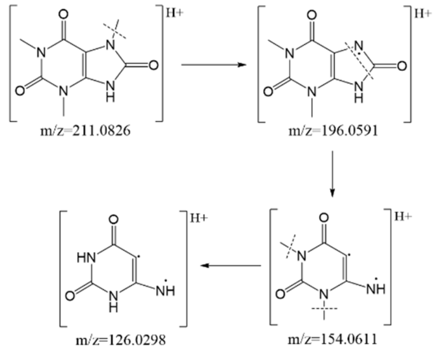

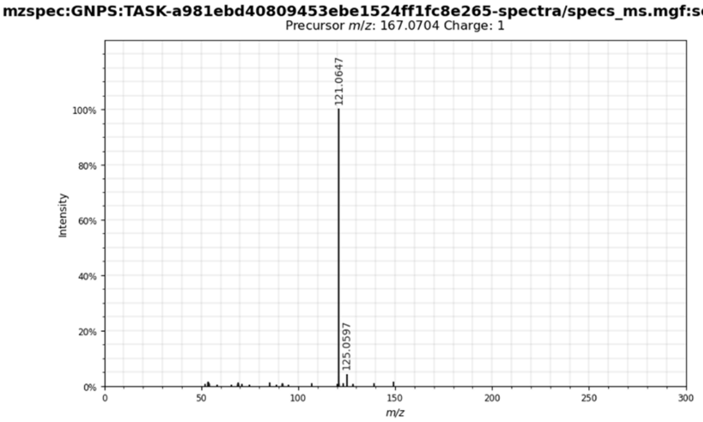

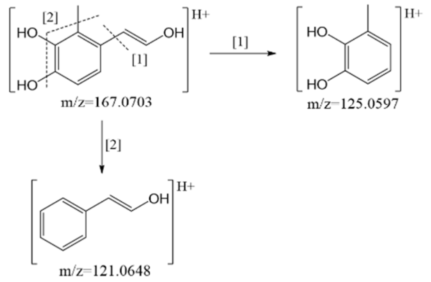

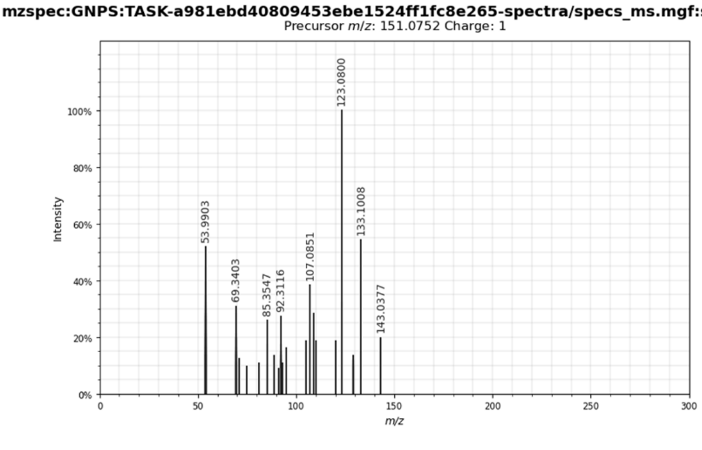

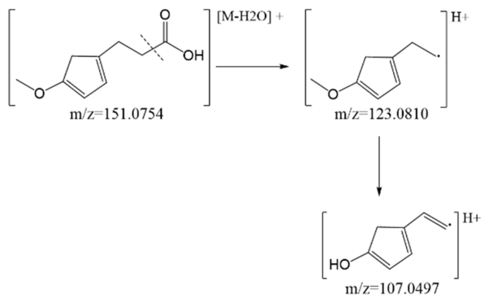

Figure S7 – Continued.

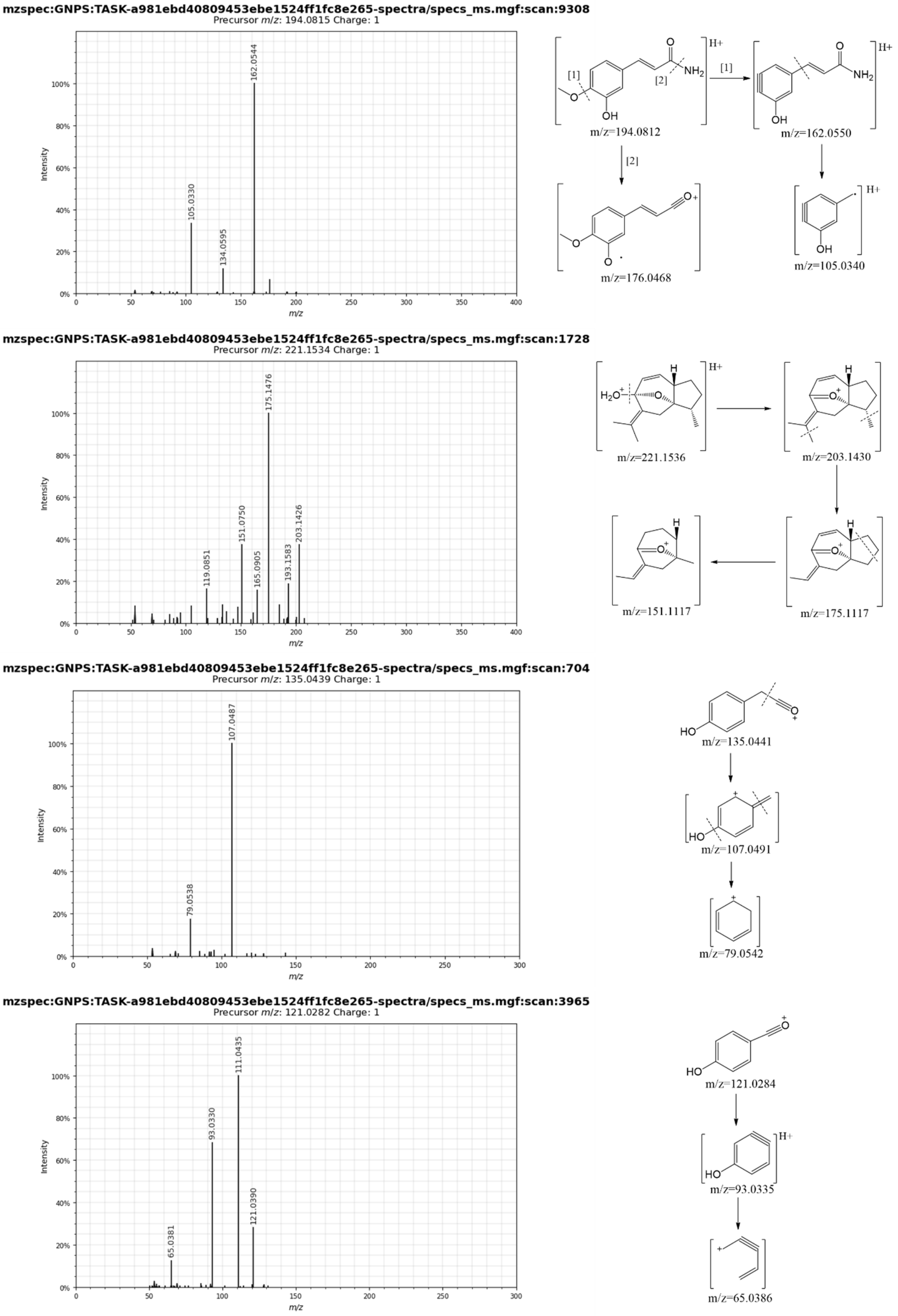

Figure S7 – Continued.
